# Whole-Transcriptome Analysis of Preadipocyte and Adipocyte and Construction of Regulatory Networks to Investigate Lipid Metabolism in Sheep

**DOI:** 10.3389/fgene.2021.662143

**Published:** 2021-07-29

**Authors:** Cheng Xiao, Tian Wei, Li Xiang Liu, Jian Qiang Liu, Chun Xin Wang, Zhi Yu Yuan, Hui Hai Ma, Hai Guo Jin, Li Chun Zhang, Yang Cao

**Affiliations:** ^1^Jilin Academy of Agricultural Sciences, Gongzhuling, China; ^2^College of Animal Science and Technology, Jilin Agricultural University, Changchun, China

**Keywords:** whole-transcriptome analysis, adipocyte differentiation, preadipocyte, mature adipocyte, differentially expressed RNAs, regulatory networks, sheep

## Abstract

Many local sheep breeds in China have poor meat quality. Increasing intramuscular fat (IMF) content can significantly improve the quality of mutton. However, the molecular mechanisms of intramuscular adipocyte formation and differentiation remain unclear. This study compared differences between preadipocytes and mature adipocytes by whole-transcriptome sequencing and constructed systematically regulatory networks according to the relationship predicted among the differentially expressed RNAs (DERs). Sequencing results showed that in this process, there were 1,196, 754, 100, and 17 differentially expressed messenger RNAs (mRNAs), long non-coding RNAs (lncRNAs), microRNAs (miRNAs), and circular RNAs (circRNAs), respectively. Gene Ontology analysis showed that most DERs enriched in Cell Part, Cellular Process, Biological Regulation, and Binding terms. Kyoto Encyclopedia of Genes and Genomes (KEGG) analysis found that the DERs primarily focused on Focal adhesion, phosphoinositide 3-kinase (PI3K)-Akt, mitogen-activated protein kinase (MAPK), peroxisome proliferator-activated receptor (PPAR) signaling pathways. Forty (40) DERs were randomly selected from the core regulatory network to verify the accuracy of the sequence data. The results of qPCR showed that the DER expression trend was consistent with sequence data. Four novel promising candidate miRNAs (miR-336, miR-422, miR-578, and miR-722) played crucial roles in adipocyte differentiation, and they also participated in multiple and important regulatory networks. We verified the expression pattern of the miRNAs and related pathways’ members at five time points in the adipocyte differentiation process (0, 2, 4, 6, 8, 10 days) by qPCR, including miR-336/ACSL4/LncRNA-MSTRG71379/circRNA0002331, miR-422/FOXO4/LncRNA-MSTRG54995/circRNA0000520, miR-578/IGF1/LncRNA-MSTRG102235/circRNA0002971, and miR-722/PDK4/LncRNA-MSTRG107440/circ RNA0002909. In this study, our data provided plenty of valuable candidate DERs and regulatory networks for researching the molecular mechanisms of sheep adipocyte differentiation and will assist studies in improving the IMF.

## Introduction

Small Tail Han sheep, a Chinese endemic breed, possesses high fecundity and strong resistance (Miao et al., 2016). Farmers and breeding enterprises widely raise Small Tail Han sheep to provide for the local consumers in Northeast China. Unfortunately, Small Tail Han sheep can no longer adapt to the current market because it has a slow growth rate and low meat quality (Kashan et al., 2005). Therefore, it is urgent to improve the quality of mutton. Intramuscular fat (IMF) content is closely related to meat quality traits and affects the taste and tenderness of mutton, changes the nutritional composition, and directly affects consumers’ purchasing decisions (Zhao et al., 2015). Therefore, increasing the content of IMF is the key to improving meat quality. Many factors affect the accumulation of IMF content including nutrition (Scollan et al., 2017), environment (Lambe et al., 2021), animal breed (Martins et al., 2020), age (Rahemi et al., 2015), gender (Gálvez et al., 2018), and genetic factors (Park et al., 2018). Although excessive caloric intake without a rise in energy expenditure increases IMF (Tang and Lane, 2012), different individuals possess the diverse ability to increase IMF in the same diet condition. The accumulation of IMF is the result of *de novo* lipogenesis and adipocyte differentiation, so when increasing IMF, we need to explore the mechanism of adipocyte differentiation.

Intramuscular adipose contains a high percentage of mature adipocytes, some preadipocytes, and a few other cells (Géloën et al., 1989). Adipocyte differentiation is a process that the preadipocyte differentiates into the mature adipocyte. The regulatory mechanisms of adipocyte differentiation are complex, and many genes (Ntambi and Young-Cheul, 2000), transcription factors (Liu et al., 2020), proteins (Gregoire et al., 1998), and hormones are involved (Obregon, 2008). Genetic factors can affect the accumulation of IMF by regulating *de novo* lipogenesis and adipocyte differentiation (Koutnikova and Auwerx, 2001). Recently, non-coding RNAs that contain long non-coding RNA (lncRNA), circular RNA (circRNA), and microRNA (miRNA) may play a core role in lipid metabolism and adipocyte differentiation (Zeng et al., 2018). LncRNA has more tissue specificity, binds to target genes by cis or trans methods (Tafer and Hofacker, 2008), and develops into pre-miRNA (Friedlander et al., 2012). LncRNA participates in various physiological functions, such as dose compensation effect, epigenetic regulation, cell cycle, and differentiation regulation (Xiao et al., 2015; Xiong et al., 2018). Some studies have reported that lncRNA plays a core role in adipocyte differentiation (Lopez-Pajares, 2016). circRNA, covalently closed by 3-terminal and 5-terminal RNAs, is more stable and conserved than other RNAs (Jeck and Sharpless, 2014). A part of circRNAs can transcribe as the protein; other positions are in intron sequences of the cell nucleus (Li Z. et al., 2015). circRNA may become potential biological markers involved in adipocyte differentiation (Yu et al., 2021). miRNA is a crucial regulatory molecule and can bind to the untranslated region (UTR) of messenger RNAs (mRNAs) to regulate their expression (Ali et al., 2020). miRNA participates in many biological functions, such as cellular growth and differentiation (Lu and Rothenberg, 2018). Many reports have identified that miRNA acts as a vital factor affecting adipocyte differentiation (Shi et al., 2016). Compete endogenous RNA (ceRNA) is a common physiological mechanism that lncRNA and circRNA competitively combine with miRNA (Thomson and Dinger, 2016). circRNA acts as a sponge to absorb miRNA competing with other RNAs (Memczak et al., 2013). Some studies have proven that ceRNA widely exists in adipocyte differentiation and forms the crucial regulatory networks (Xu et al., 2015; Chen et al., 2018). Although many studies related to adipocyte differentiation were published, the detailed molecular mechanism remains unclear, especially non-coding RNA and ceRNA in sheep.

To explore the molecular mechanisms and identify the candidate RNAs in sheep adipocyte differentiation, we compared the changes between preadipocytes and mature adipocytes by whole-transcriptome analysis. Our data provided many novel candidate RNAs, ceRNA networks, and, at the same time, research directions and theoretical basis for related research. This work is meaningful for excavating the molecular mechanism of sheep adipocyte differentiation and increasing IMF.

## Materials and Methods

### Preadipocyte Isolation, Culture, and Differentiation

The Small-Tailed Han sheep was raised as experimental animals in the Jilin Academy of Agricultural Sciences. A 2-month-old healthy male sheep’s groin adipose tissue was isolated to extract the preadipocytes. The tissue was washed with ice-cold phosphate-buffered saline (PBS; Sigma-Aldrich, St. Louis, MO, United States) containing 1% penicillin/streptomycin (Sigma) and cut into small pieces, then the tissue blocks were digested for 1 h using collagenase Type II (Sigma) and 0.25% trypsin (Sigma) to collect preadipocytes. The cells were cultured with a complete culture medium containing 10% fetal bovine serum (Gemini Bio-Products, Woodland, CA, United States), 1% penicillin/streptomycin (Sigma), and DMEM-F12 medium (Sigma) in a 37°C and 5% CO_2_ incubator (Thermo Fisher Scientific, Waltham, MA, United States) and replaced with new culture medium every 48 h. When the cells are overgrown in the culture dish, they are induced to differentiate into mature adipocytes by the exogenous inducer. The cells were treated with inducer I solution containing 10 mg/ml insulin (Sigma), 1.0 mM dexamethasone (Sigma), 0.5 mM IBMX (Sigma), and complete culture medium for 48 h, inducer II solution containing 10 mg/ml insulin (Sigma) and complete culture medium for 48 h, and then fresh complete culture medium to continue cell culture. The whole process of cell differentiation underwent about 12 days.

### Oil Red O Staining

Oil red O staining solution can identify adipocytes because the lipid droplets within the cell can be stained red, but other cells cannot. Oil red O staining occurs on the 12th day when the cells are growing, which is the eighth day when the cells start to differentiate. The adipocytes were washed with PBS buffer three times and fixed with 4% paraformaldehyde solution (Sangon Biotech Co., Ltd., Shanghai, China) in a 37°C and 5% CO_2_ incubator for 30 min. The cells were washed with PBS buffer three times, then cells were treated with Oil red O (Sigma) for 30 min in the incubator. After this, the cells were washed with PBS buffer three times, the staining was observed with a microscope, and pictures were taken.

### RNA Extraction and Qualification

The fourth-generation preadipocytes possess fast and stable growth and can be used as experimental cells based on previous experimental experience. Some preadipocytes are regarded as group P, and the remaining cells differentiate into adipocytes as group M. Each group has three repetitions (technical repetition); they are P1, P2, P3 and M1, M2, M3. The cells’ total RNA was extracted using TRIzol reagent (Thermo Fisher Scientific, Waltham, MA, United States) according to the instructions, then RNA degradation and contamination were monitored using 1% agarose gels. NanoPhotometer spectrophotometer (IMPLEN, CA, United States) assesses the purity of RNA. Qubit RNA Assay Kit in a Qubit 2.0 Fluorometer (Life Technologies, CA, United States) measured the concentration of RNA. Nano 6000 Assay Kit in a Bioanalyzer 2100 system (Agilent Technologies, CA, United States) evaluated the integrity of RNA (Ma et al., 2019).

### Library Preparation

Here, 3 μg total RNA of each sample was used to construct the lncRNA library. The total RNA was digested by DNase I, and ribosomal RNA (rRNA) was removed using Ribo-Zero^TM^ Gold Kit (Illumina, San Diego, United States); at this time, the lncRNA library contains mRNA. Since mRNA contains a specific Poly-A tail, oligo(dT) magnetic beads can specifically bind to the Poly-A tail to separate mRNA. Fragmentation Buffer was added to the reaction system to fragment RNA into short fragments (about 200–500 nt), and then the fragments were used as templates to synthesize the first strand of cDNA with six-base random hexamer primers, and then buffer, dNTPs, RNase H, and DNA Polymerase I were used to synthesize the second strand of cDNA. QiaQuick PCR kit purified cDNA and EB buffer resolved end reparation and single nucleotide A (adenine) addition and adaptor. The target fragment is recovered by agarose gel electrophoresis, the uracil-N-glycosylase (UNG) enzyme degraded the second strand of cDNA, and the suitable fragments were selected as templates for PCR amplification. Finally, agarose gel electrophoresis recovers the target fragments to construct the library. To construct a circRNA library, it is necessary to add an RNase enzyme to remove linear RNA after rRNA is removed, so that circRNA can be obtained. After the RNA of the sample is qualified, the Small RNA Sample Pre Kit constructs the miRNA library; small RNA sequence length is small and has a special structure at the 3′ and 5′ ends (the 5′ end has a complete phosphate group, and the 3′ end has a hydroxyl group). The small RNA is directly connected to both ends with an adaptor, and then reverse transcription is used to synthesize cDNA. Then, after PCR amplification, polyacrylamide gel electrophoresis (PAGE) was performed to separate the target DNA fragments; the cDNA library is recovered by cutting the gel. The constructed library was sequenced using an Illumina system (Agilent Technologies Inc.) by Annoroad Gene Technology Co., Ltd. (Beijing, China).

### Raw Data Processing

The original results of Illumina high-throughput sequencing are image data files, which are converted into Raw Reads after Base Calling by bcl2fastq2 software, and the results are stored in the FASTQ (referred to as fq) file format. In the FASTQ format file, each base corresponds to a base quality character, and the ASCII code value corresponding to each base quality character minus 33 (Sanger quality value system) is the sequencing quality score of the base (Phred Quality Score). Different Phred Quality Scores represent different base sequencing error rates. For example, Phred Quality Score values of 20 and 30 indicate base sequencing error rates of 1 and 0.1%, respectively. Raw data contain sequencing adapter sequences and low-quality sequences. To ensure the quality of the information analysis data, we filter raw data sequences and use Cutadapt and fastx_toolkit to remove the adaptor, low-quality reads, reads with an N content greater than 5%, and reads that match ribosomal RNA.

### Comparison Analysis and Mapped Reads Assembly

To better identify lncRNAs and mRNAs, clean RNA sequencing (RNA-Seq) reads were mapped to the genome using Hisat2. To identify circRNAs, clean RNA-Seq reads were mapped to the genome using the BWA-MEM algorithm. BWA-MEM can quickly and efficiently compare reads with the genome. For the accuracy of subsequent miRNA analysis, clean miRNA-Seq reads were mapped to the genome using Bowtie software (setting allows one mismatch). The reference genome and gene annotation files were downloaded from the NCBI database. Version: Ovis_aries.NCBI.GCF_002742125.1.v1.0. GCF_002742125.1_Oar_rambouillet_v1.0_genomic. gff.gz. StringTie can quickly assemble transcripts using RNA-Seq alignment results. Reads were divided into different categories, and built splice graph to determine the transcript. Each transcript build flow network for path of heaviest coverage to compute maximum flow to evaluate abundance, and combine the complex data set assemble into a transcript.

### Long Non-coding RNA Identification

We mainly screen for three types: long intergenic non-coding RNA (lincRNA), intronic lncRNA, and antisense lncRNA. The basic screening conditions are as follows: (1) The length of the transcript is greater than or equal to 200 bp, and the number of exons is greater than or equal to 2; (2) Calculating the read coverage of each transcript, and transcripts with less than five reads in all samples were removed; (3) gffcompare^1^ compares the annotation files of the species to screen out the known mRNA and other non-coding RNA (rRNA, tRNA, snoRNA, snRNA, etc.); (4) Identifying potential lincRNA, intronic lncRNA, and antisense lncRNA according to the class_code information (“u,” “i,” “x”) in the comparison result. Through the initial screening of lncRNA in the previous step, a variety of coding potential analysis software is used to screen, mainly Coding-Non-Coding Index (CNCI), Coding Potential Calculator (CPC), Coding Potential Assessment Tool (CPAT for animals only), and PFAM protein domain analysis. Several analysis methods all distinguish the non-coding transcript as the final novel lncRNA data set.

### Circular RNA Identification

The current research mainly is on exonic circular RNA (ecircRNA) and the cyclization mechanism: (1) lariat-driven circularization (2) intron pairing-driven circularization. The difference between the two models is whether the first step of cyclization is to form a lariat-driven structure or the introns on both sides of the exon are complementarily paired first. No matter which form it is, it is formed by trans-splicing the splice donor (SD) of the downstream exon of the circRNA and the splice acceptor (SA) of the upstream exon. The main idea of recognizing circRNA: looking for the GT-AG signal next to the junction site. CIRI (Gao et al., 2015) is an efficient and fast circRNA identification tool. BWA-MEM algorithm is used to split and compare the sequences, and then the resulting SAM file is scanned to find paired chiastic clipping (PCC) and paired-end mapping (PEM) sites, as well as GT-AG splicing signals. The sequence with junction site was realigned with a dynamic programming algorithm to ensure the reliability of identifying circRNA.

### miRNA Identification

According to the mapped reads, it is compared with the specified species sequence in the miRBase database to identify known miRNAs. Clean Reads that are not annotated as known miRNAs are compared with the non-coding RNA sequence in Rfam (13.0) to realize the annotation of rRNA, tRNA, snRNA, snoRNA, and other non-coding RNAs. For Clean Reads that are not annotated as known miRNA, non-coding RNA, and Repeat, the small RNA derived from mRNA is annotated by matching with the location information of gene exons and introns (100% position overlap). There are still some features of miRNAs, but no sequences have been discovered so far, so novel miRNA predictive analysis is needed. The software miRDeep2 is used to predict novel miRNAs, obtain the matched Clean Reads information of each novel miRNA, and obtain the structure and expression information of each novel miRNA.

### Analysis of Differentially Expressed RNAs

The expression levels of the protein-coding genes and lncRNAs were estimated by FPKM (Fragments Per Kilobase of transcript per Million mapped reads), which is a very effective tool for quantitatively estimating gene expression and can eliminate the influence of the difference in gene length and sequencing amount on the calculated gene expression, and the results can be directly used to compare the expressed difference between different samples. Due to the particularity of circRNA, it is difficult to accurately obtain the information of circRNA Reads on all alignments (linear RNA interference), so the usual expression estimation method is to use the number of Junction reads to estimate the expression of circRNA. We selected SRPBM (Spliced Reads per Billion Mapping) (Li Y. et al., 2015) normalization method to quantify the expression of circRNA. TPM (Transcripts Per Million) is used to estimate the expression level of miRNA, which is calculated based on the reads (incompletely mature or degraded) that are aligned to the miRNA precursor and slide in a certain area of the mature body. We selected DEseq2 to analyze the differential expression of RNAs, comparing the treatment group and the reference group. |log2Ratio| ≥ 1 and *p* < 0.05 were set as the threshold in the differential expression analysis.

### Target RNA Prediction

LncRNA binds to target genes in Cis or Trans mode. Cis binding method prediction: the protein-coding genes adjacent to lncRNA (upstream and downstream 50 kb) will be screened out as target genes. Trans combination method prediction: when the number of samples is greater than or equal to 6, the target gene is screened according to the correlation coefficient between the expression of lncRNA and mRNA (correlation coefficient ≥ 0.9). The forecasting software is implemented using Annoroad’s in-house scripts. There are multiple binding sites of miRNA on the circRNA sequence. When miRNA is adsorbed, it cannot regulate its corresponding target gene, thus acting as a sponge of miRNA molecules, and then we selected miRanda (3.3a) (Enright et al., 2003) for target prediction. MiRanda (Enright et al., 2003) and TargetScan (Agarwal et al., 2015), two target prediction software, were selected to predict miRNA target genes, and the intersection of the two target prediction results is taken as miRNA target genes.

### Gene Ontology and Kyoto Encyclopedia of Genes and Genomes Pathway Analysis

GOseq R package (ver. 2.12) conducted Gene Ontology (GO) analysis according to the following rules: differentially expressed genes (DEGs), differentially expressed circular RNA (DEcircRNA)-deriving genes, target genes of differentially expressed long non-coding RNAs (DElncRNAs), and differentially expressed miRNAs (DEmiRNAs). GO analysis was used to annotate the genes with terms under biological process, cellular component, and molecular function categories. KOBAS software (2.0) conducted Kyoto Encyclopedia of Genes and Genomes (KEGG) pathways analysis according to the same rules. GO and KEGG terms featuring *p* < 0.05 were considered significantly enriched.

### Regulatory Network Construction

According to the prediction results of target RNAs, we systematically constructed various regulatory networks of the differentially expressed RNAs (DERs) related to sheep adipocyte differentiation. The two-element network was based on the following relationships: target mRNA of lncRNA, target mRNA of miRNA, lncRNA can become miRNA, circRNA adsorbs miRNA. Then, we constructed ceRNA networks, including the three-element network and four-element network based on various RNAs that can competitively bind the same miRNA response elements (MREs). Cytoscape 3.6.1 visualized the networks.

### Quantitative Real-Time PCR Validation

We randomly selected 40 DERs from the regulatory networks to identify the accuracy of sequencing results using qPCR and also explored the expression trend of the RNAs at five time points during the preadipocyte to mature adipocyte period (2, 4, 6, 8, 10 days). The glyceraldehyde-3-phosphate dehydrogenase gene (*Gapdh*) and U6 were internal controls. The total RNA transcribed into the cDNA using a reverse transcription kit (TaKaRa, Japan) as qPCR template, and the glyceraldehyde-3-phosphate dehydrogenase gene (Gapdh) and U6 were internal controls for qPCR. The primer sequences were synthesized by Genewiz Company as Supplementary Tables 1–4. qPCR was performed on LightCycler Roche 480 (Roche) with SuperMix Real PreMix Plus (SYBR green) (Roche). The delta-delta CT method calculated the relative expression level of RNAs.

### Statistical Analysis

The sequencing data of samples were compared according to the following three groups: M1 vs. P1, M2 vs. P2, and M3 vs. P3. The commonly owned data among the three groups were the final results of the adipocyte differentiation process. R software calculated parameters for the high-throughput sequencing-related data and differential expression analyses. All data representation using means ± SD and a *p*-value threshold of 0.05 was to infer statistically significant expression changes.

## Results

### Adipocyte Culture and Differentiation

**FIGURE 1:**
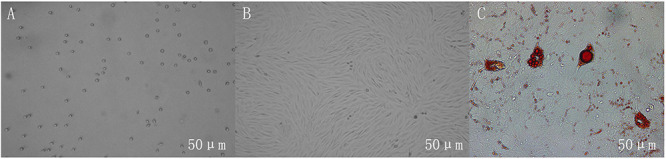
Growth morphology of adipocytes in different periods. **(A)** Non-adherent cells are spherical. **(B)** The adherent cells are irregularly shaped, and the cells are photographed on the fourth day of culture. **(C)** The lipid droplets in differentiated adipocytes can be stained red by Oil Red O, and the cells are photographed on the 12th day of culture. Scales bar: 50 μm.

Adipocytes’ differentiation process mainly undergoes two stages: proliferation and differentiation. As shown in Figure 1, the non-adherent preadipocytes are spherical with a diameter of 10–30 μm. The cells begin to adhere to the wall and become irregular fusiform after 6 h. When the cells are overgrown in the culture dish, the exogenous inducer promotes the differentiation of the cells into mature adipocytes. Under the microscope, it can be seen that there are lipid droplets inside the cells, and the lipid droplets can be stained by Oil Red O, which indicates that the cells are successfully differentiated.

### Overview of RNA Sequencing

As shown in Table 1, the quality of the sequencing data is high, the proportion of high-quality clean reads exceeds 95%, and the average percentage of Q30 is above 93%. The clean reads mapping rates of lncRNA and mRNA were more than 93%. We obtained 18,968 RNA transcripts, of which 1,713 known and 5,640 novel lncRNAs were in the P1 group, 1,705 known and 5,640 novel lncRNAs were in the P2 group, 1,680 known and 5,637 novel lncRNAs were in the P3 group, 1,569 known and 5,548 novel lncRNAs were in the M1 group, 1,611 known and 5,537 novel lncRNAs were in the M2 group, and 1,666 known and 5,602 novel lncRNAs were in the M3 group. The mapping rates of circRNA surpass 99% (Table 1). We identified 3,480 circRNAs, of which the CLASSIC circRNAs account for more than 80%. The mapping rates of miRNA exceed 97% (Table 2). We identified 150 known and 326 novel miRNAs in the P1 group, 150 known and 378 novel miRNAs in the P2 group, 150 known and 375 novel miRNAs in the P3 group, 151 known and 372 novel miRNAs in the M1 group, 149 known and 361 novel miRNAs in the M2 group, and 150 known and 342 novel miRNAs in the M3 group.

**TABLE 1:** Alignment and quantification statistics in each sample RNA-Seq library.

**Sample**	**P1**	**P2**	**P3**	**M1**	**M2**	**M3**
Raw reads	70,089,738	70,608,836	89,908,418	83,662,284	84,682,784	83,821,350
Clean reads	67,227,174	67,721,360	86,430,498	80,025,662	81,211,530	80,248,416
Clean Reads rate (%)	95.92	95.91	96.13	95.65	95.9	95.74
Clean Q30 rate (%)	93.39	93.42	94.14	94.37	93.99	94.36
LncRNA and mRNA mapping rate (%)	95.89	96	96.15	93.32	95.86	96.16
circRNA mapping rate (%)	99.99	1	1	99.97	1	1

*circRNA, circular RNA; lncRNA, long non-coding RNA; mRNA, messenger RNA; RNA-Seq, RNA sequencing.*

**TABLE 2:** Alignment and quantification statistics in each sample miRNA library.

**Sample**	**P1**	**P2**	**P3**	**M1**	**M2**	**M3**
Total reads	22,413,923	18,892,622	18,752,504	24,882,694	25,413,485	20,585,959
Perfect match reads	18,543,399	15,886,883	15,820,075	21,054,917	21,678,813	17,451,683
Match rate (%)	97.45	97.72	97.86	97.76	97.99	97.8
Not match rate (%)	2.55	2.28	2.14	2.24	2.01	2.2

### Analysis of Differentially Expressed RNAs

**FIGURE 2:**
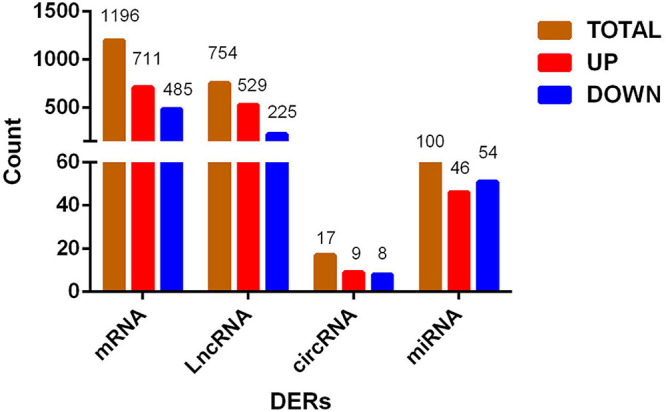
Histogram of differentially expressed RNA shared by three groups. From preadipocytes to mature adipocytes, brown column is the number of total differentially expressed RNAs, red column is RNAs with increased expression, and blue column is RNAs with decreased expression.

Using |log2Ratio| ≥ 1 and *p* < 0.05 as the criteria for screening DERs, the volcano chart shows the number of DERs in each group (Supplementary Figure 1). R language draws a cluster map to reflect the expression changes of DERs under different conditions (Supplementary Figure 2). DERs of each group are different, and we use the three groups of shared DERs as the final data. Figure 2 showed that we identified 1,196 DEGs, of which 711 genes were upregulated, and 485 were downregulated. There were 754 DElncRNAs in the process, of which 529 lncRNAs were upregulated, and 225 lncRNAs were downregulated. There were 17 DEcircRNAs in the process, of which 9 circRNAs were upregulated, and 8 circRNAs were downregulated. In addition, 100 DEmiRNAs dysregulated, 46 miRNAs were upregulated, and 54 miRNAs were downregulated between the P and M groups. The DERs’ detailed information is in Supplementary Tables 5–8.

### Gene Ontology and Kyoto Encyclopedia of Genes and Genomes Pathway Analysis

GO analysis showed that the DERs were mainly involved in the cell part of the cellular component classification. Many DERs belonged to cellular processes and biological regulation on the physiological process classification. The molecular function assessment showed that the DERs associated with binding (Supplementary Figure 3). KEGG pathway analysis found that DEGs mainly enriched in the Focal adhesion, Axon guidance, and phosphoinositide 3-kinase (PI3K)-Akt signaling pathway. Peroxisome proliferator-activated receptor (PPAR), adipocytokine, and mitogen-activated protein kinase (MAPK) signaling pathways were also significantly enriched, but there was no significant enrichment in Fatty acid biosynthesis, Insulin signaling pathway, fat digestion and absorption, and fatty acid metabolism. The top three lncRNAs were MAPK signaling pathway, endocytosis, and pathways in cancer. Insulin and adipocytokine signaling pathway and fatty acid metabolism enriched significantly, but there was no significant enrichment in fat digestion and absorption, fatty acid biosynthesis, and PPAR signaling pathways. Most circRNAs focused on focal adhesion, FoxO signaling pathway, longevity regulating pathway-worm, axon guidance, and PI3K–Akt signaling pathways. A large number of miRNAs were involved in focal adhesion and PPAR signaling pathways (Supplementary Figure 4).

### Construction of Regulatory Networks

#### Two-Element Network

**FIGURE 3:**
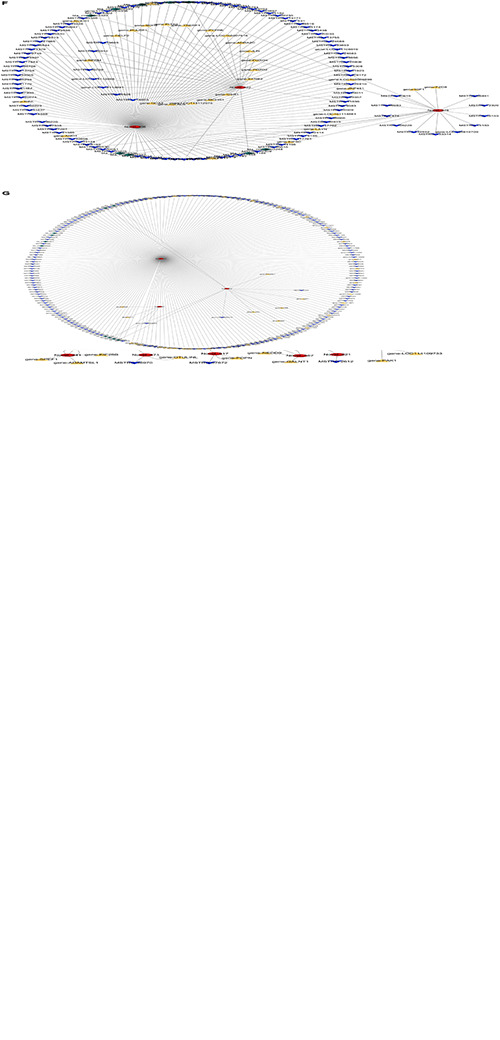
Regulatory networks from preadipocytes to mature adipocytes. **(A)** Circular RNA (circRNA)–microRNA (miRNA) network regulation diagram; the red graphic is miRNA, and the green graphic is circRNA. **(B)** miRNA–messenger RNA (mRNA) network regulation diagram; the yellow graphic is mRNA. **(C)** Long non-coding RNA (lncRNA)–miRNA–mRNA network regulation diagram; the blue graphic is mRNA. **(D)** circRNA–miRNA–mRNA network regulation diagram between M1 vs. P1 and M2 vs. P2 groups. **(E)** circRNA–miRNA–mRNA network regulation diagram between M2 vs. P2 and M3 vs. P3 groups. **(F)** LncRNA–circRNA–miRNA–mRNA network regulation diagram between M1 vs. P1 and M2 vs. P2 groups. **(G)** LncRNA–circRNA–miRNA–mRNA network regulation diagram between M2 vs. P2 and M3 vs. P3 groups.

A lncRNA combines with multiple target genes, at the same time, a gene can also be targeted by multiple lncRNAs in the lncRNA–mRNA networks. There were 729 DElncRNAs and 1,844 DE target genes in the three groups. There were 46 DElncRNAs and 24 target miRNAs in the lncRNA–miRNA networks. The lncRNA–mRNA and lncRNA–miRNA regulatory networks were too complex and huge, so the networks were shown clearly in a chart on the manuscript. Detailed information is shown in Supplementary Tables 9, 10. Figures 3A,B showed circRNA–miRNA and miRNA–mRNA regulatory networks. Detailed information is shown in Supplementary Tables 11, 12.

#### Three-Element Network

As shown in Figure 3C, 13 novel miRNAs play a central role in the mRNA–miRNA–lncRNA networks. Detailed information is shown in Supplementary Table 13. In miRNA–mRNA–circRNA networks, we did not find shared networks in the three groups, but there are shared networks in M1 vs. P1 and M2 vs. P2, and there are other shared networks in M2 vs. P2 and M3 vs. P3. There are six novel miRNAs (miR-336, miR-422, miR-578, miR-722, miR-748, and miR-831) that may play a core role in the regulatory networks (Figures 3D,E). Detailed information is shown in Supplementary Table 14.

#### Four-Element Network

In miRNA–mRNA–circRNA–lncRNA networks, we did not find shared networks in the three groups, but there are shared networks in M1 vs. P1 and M2 vs. P2, and there are other shared networks in M2 vs. P2 and M3 vs. P3 (Figures 3F,G). Interestingly, six novel miRNAs (miR-336, miR-422, miR-578, miR-722, miR-748, and miR-831) can simultaneously form three-element and four-element networks. Detailed information is shown in Supplementary Table 15.

### Screening Out Candidate RNAs and Core Networks

Because the number of DERs and networks was large and complex, we screened out the highest expression DERs and core networks. The highest expression of DEGs was screened out based on | logFC| > 5. The highest expression of lncRNAs and circRNAs was chosen based on | logFC| > 2 because the | logFC| value of DElncRNAs and microRNAs is low (Table 3). The | logFC| value of DEmiRNAs is high, so the highest expression of miRNAs was selected based on | logFC| > 10. miRNAs’ detailed information is shown in Table 4. The raw data of sequencing have been in the public database (Sequence Read Archive): SUB7601005 and SUB7600133^2,^^3^.

**TABLE 3:** The highest expression of DERs in M group vs. P group of RNA-Seq.

**Description**	**Log2(M/P group)**	**Regulation**	***p*-value**	**Position**	**Biotype**
SPINK5	−5.214585327	Down	4.36E−175	chrNC_040256.1:61843953−61931728:+	Protein coding
SLITRK6	−5.742358445	Down	5.89E−62	chrNC_040261.1:66253981−66260610:−	Protein coding
LOC101106199	−6.537740726	Down	2.82E−22	chrNC_040266.1:52094769−52096364:+	Protein coding
SLC9A3	−6.122703226	Down	2.68E−17	chrNC_040267.1:78089680−78123950:+	Protein coding
LOC106990846	−2.073319757	Down	1.01E−89	chrNC_040252.1:67232773−67237128:+	lncRNA
LOC114116841	−2.315348304	Down	0.00094041	chrNC_040262.1:20528946−20533678:+	lncRNA
LOC105602850	−2.367815724	Down	0.003113683	chrNC_040268.1:57249831−57306438:−	lncRNA
LOC114113974	−2.730385804	Down	0.036100724	chrNC_040252.1:4213089−4240720:−	lncRNA
LOC114114545	−2.647923643	Down	0.000519611	chrNC_040255.1:103110999−103142205:+	lncRNA
LOC105604950	−3.845863021	Down	0.000341618	chrNC_040276.1:17541786−17605265:−	lncRNA
LOC105607182	−2.367815724	Down	2.90E−05	chrNC_040253.1:38409442−38410287:−	lncRNA
LOC114110446	−5.145423303	Down	2.20E−05	chrNC_040274.1:5639080−5641719:+	lncRNA
MSTRG.80212	−5.052313898	Down	6.67E−09	chrNC_040262.1:35134262−35138700:+	lncRNA
MSTRG.105803	−4.845863021	Down	0.000147779	chrNC_040268.1:48523635−48527825:+	lncRNA
MSTRG.121783	−5.730385804	Down	1.69E−07	chrNC_040273.1:14918722−14956227:+	lncRNA
bta_circ_0000680	−4.229703279	Down	0.004356129	chrNC_040253.1:777960920.77797217:−	circRNA

*circRNA, circular RNA; DER, differentially expressed RNA; lncRNA, long non-coding RNA; RNA-Seq, RNA sequencing.*

**TABLE 4:** The highest expression of DEmiRNAs in M group vs. P group of RNA-Seq.

**Description**	**Log2(M/P group)**	**Regulation**	***p*-value**	**Sequence**	**Biotype**
Novel_220	−15.12252412	down	0	ACCACAGGGTAGAACCACGGAC	miRNA
Novel_455	−14.97920218	down	0	CTCAGTCAGCCTTGTGGATGTA	miRNA
Novel_90	−14.53355481	down	0	AAAAGCTGGGTTGAGAGGGCGA	miRNA
Novel_172	−13.97693693	down	0	AACTGTTTGCAGAGGAAACTGAG	miRNA
Novel_169	−13.23737812	down	0	AACTGGCCCACAAAGTCCCGCT	miRNA
Novel_362	−13.0942399	down	0	CAAGTCACTAGTGGTTCCGTTTAGT	miRNA
Novel_890	−11.86052381	down	6.70E−207	TTATCAGAATCTCCAGGGGTACT	miRNA

*DEmiRNA, differentially expressed microRNA; miRNA, microRNA; RNA-Seq, RNA sequencing.*

The core networks were screened out based on three- and four-element networks, and they all contained four crucial novel miRNAs (miR-336, miR-422, miR-578, and miR-722). The miRNAs can form various networks because they had many targets, such as the following: miR-336 has 402 target RNAs (58 genes, 328 lncRNAs, 16 circRNAs); miR-422 has 24 target RNAs (11 genes, 11 lncRNAs, 2 circRNAs); miR-578 has 335 targets (46 genes, 273 lncRNAs, 16 circRNAs); miR-722 has 201 target RNAs (24 genes, 163 lncRNAs, 14 circRNAs). We considered the miRNAs as the candidate miRNAs. Detailed information is shown in Table 5.

**TABLE 5:** Core miRNAs of important networks in M group vs. P group of RNA-Seq.

**Description**	**Log2(M/P group)**	**Regulation**	***p*-value**	**Sequence**	**Biotype**
Novel_366	−6.315266783	Down	4.05E−10	CACAGCTCCAGGGGATGCCGTTC	miRNA
Novel_422	−6.76272576	Down	4.66E−13	CCTCCCCTTCCTCCCTCCCTCCC	miRNA
Novel_578	−4.971312382	Down	9.65E−05	GGGTTTCCCTGGTGGCTCAGA	miRNA
Novel_722	−9.988908241	Down	2.23E−72	TCGCTCAGTCGTGTCCGACTCT	miRNA
Novel_748	−5.525383867	Down	4.38E−06	TCTGGAGGGGCAGAAGGAGAAGC	miRNA
Novel_831	1.057978306	Up	7.77E−95	TGGCAGTGTCTTAGCTGGTTGTTG	miRNA
Novel_842	−5.110346368	Down	7.73E−05	TGGGCGGAGGTGGGGCGGGGGGCCC	miRNA

*miRNA, microRNA; RNA-Seq, RNA sequencing.*

### Validation of the Accuracy of RNA Sequencing Data by qPCR

**FIGURE 4:**
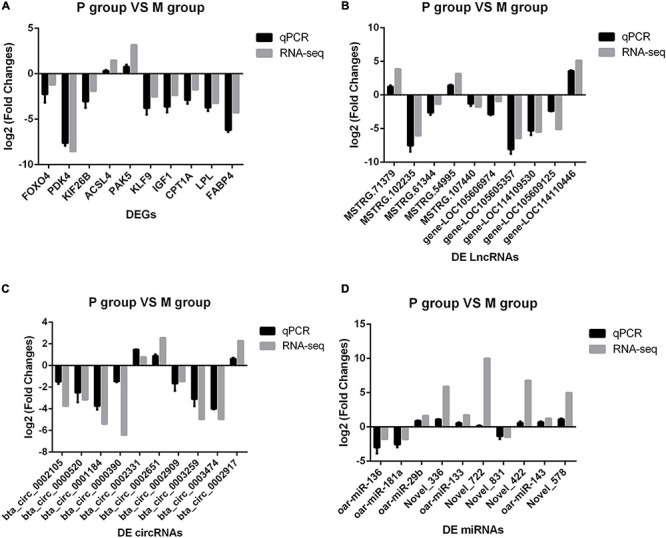
Comparison of sequencing results and qPCR results. The abscissa is differentially expressed RNAs (DERs), the ordinate is Log2(Fold Change), the gray column is the sequencing result, and the black column is the qPCR result. **(A)** Validation of messenger RNA (mRNA) results by qPCR. **(B)** Validation of long non-coding RNA (lncRNA) results by qPCR. **(C)** Validation of circular RNA (circRNA) results by qPCR. **(D)** Validation of microRNA (miRNA) results by qPCR.

To validate the accuracy of RNA-Seq data, we selected 40 DERs from the highest expression DERs and the core regulatory networks, including four well-known genes that affect the adipocyte differentiation, including Lipoprotein lipase (LPL) (Ailhaud et al., 1985), Fatty acid-binding protein 4 (FABP4) (Moseti et al., 2016), Insulin-like growth factor 1 (IGF1) (Chun et al., 2019), Carnitine palmitoyltransferase 1A (CPT1A) (Calderon et al., 2016), and novel crucial miRNAs (miR-336, miR-422, miR-578, miR-722). The qPCR results were highly consistent with the RNA-Seq data (Figure 4). It suggested that RNA-Seq data were credible and accurate. DERs’ detailed information is shown in Tables 6, 7.

**TABLE 6:** Validation DERs by qPCR.

**Description**	**Log2(M/P group)**	**Regulation**	***p*-value**	**Position**	**Biotype**
FOXO4	1.206825243	Up	2.38E−29	chrNC_040278.1:66559700−66566788:+	Protein coding
PDK4	8.51278818	Up	0	chrNC_040255.1:14709622−14723348:−	Protein coding
KIF26B	1.958753835	Up	1.40E−264	chrNC_040263.1:34375794−34974888:−	Protein coding
ACSL4	−1.484972615	Down	9.18E−274	chrNC_040278.1:134005859−134080888:+	Protein coding
PAK5	−3.164830284	Down	0.009030858	chrNC_040264.1:2315164−2741382:−	Protein coding
KLF9	2.520358431	Up	0	chrNC_040253.1:71284364−71311713:+	Protein coding
IGF1	2.340239857	Up	0	chrNC_040254.1:183945231−184028095:−	Protein coding
CPT1A	1.738046955	Up	4.08E−262	chrNC_040272.1:47903982−47963597:−	Protein coding
LPL	3.23308832	Up	3.81E−37	chrNC_040253.1:48325425−48351715:−	Protein coding
FABP4	4.280841452	Up	1.62E−10	chrNC_040260.1:62825793−62830309:−	Protein coding
bta_circ_0002105	3.747576644	Up	0.007144757	chrNC_040263.1:236713980.23815809:+	circRNA
bta_circ_0000520	3.162614143	Up	0.040244255	chrNC_040253.1:1914752360.191552199:−	circRNA
bta_circ_0001184	5.825877078	Up	3.30E−08	chrNC_040256.1:159915940.16093665:+	circRNA
bta_circ_0000390	6.410839578	Up	1.71E−11	chrNC_040253.1:1203039920.120348810:+	circRNA
bta_circ_0002331	−0.774234923	Down	5.16E−33	chrNC_040264.1:664698010.66593120:+	circRNA
bta_circ_0002651	−2.559851881	Down	4.34E−05	chrNC_040268.1:553308110.55331699:+	circRNA
bta_circ_0002909	1.452120761	Up	0.015200022	chrNC_040271.1:304809270.30573486:+	circRNA
bta_circ_0003259	4.969969066	Up	1.04E−05	chrNC_040276.1:140470320.14062601:+	circRNA
bta_circ_0003474	4.969969066	Up	1.04E−05	chrNC_040278.1:941054400.94114703:−	circRNA
bta_circ_0002917	−2.287418777	Down	5.26E−09	chrNC_040271.1:40105230.4029153:+	circRNA
MSTRG.71379	−3.853768219	Down	3.22E−41	chrNC_040260.1:50049753−50057325:+	lincRNA
MSTRG.102235	6.024501699	Up	1.16E−09	chrNC_040267.1:32049818−32061433:−	lincRNA
MSTRG.61344	1.328310243	Up	7.85E−47	chrNC_040258.1:44623533−44652963:−	Antisense
MSTRG.54995	−3.177513741	Down	0	chrNC_040257.1:26304321−26424276:+	Intronic
MSTRG.107440	1.761467293	Up	0.016201024	chrNC_040268.1:78672493−78673817:+	lincRNA
LOC105606974	0.942039538	up	0.000111223	chrNC_040253.1:258708631−258721999:−	lncRNA
LOC105605357	6.443291333	up	3.23E−87	chrNC_040278.1:12643932−12646966:−	lncRNA
LOC114109530	5.498432887	up	3.97E−07	chrNC_040271.1:52087348−52095154:+	lncRNA
LOC105609125	5.102504211	up	1.12E−05	chrNC_040257.1:63372022−63385936:−	lncRNA
LOC114110446	−5.145423303	down	2.20E−05	chrNC_040274.1:5639080−5641719:+	lncRNA

*circRNA, circular RNA; DER, differentially expressed RNA; lincRNA, long intergenic non-coding RNA; lncRNA, long non-coding RNA.*

**TABLE 7:** Validation DEmiRNAs by qPCR.

**Description**	**Log2(M/P group)**	**Regulation**	***p*-value**	**Sequence**	**Biotype**
oar-miR-136	1.782238672	Up	5.34E−29	ATCCATTTGTTTTGATGATGGA	miRNA
oar-miR-181a	1.787057294	Up	0	AACATTCAACGCTGTCGGTGAGT	miRNA
oar-miR-29b	−1.635919823	Down	0	TAGCACCATTTGAAATCAGTGT	miRNA
oar-miR-133	−1.727689283	Down	4.20E−67	TTGGTCCCCTTCAACCAGCTGT	miRNA
oar-miR-143	−1.226226546	Down	0	TGAGATGAAGCACTGTAGCTC	miRNA

*DEmiRNA, differentially expressed microRNA; miRNA, microRNA.*

### Validation Expression Trend of Differentially Expressed RNAs in the Process of Adipocyte Differentiation

**FIGURE 5:**
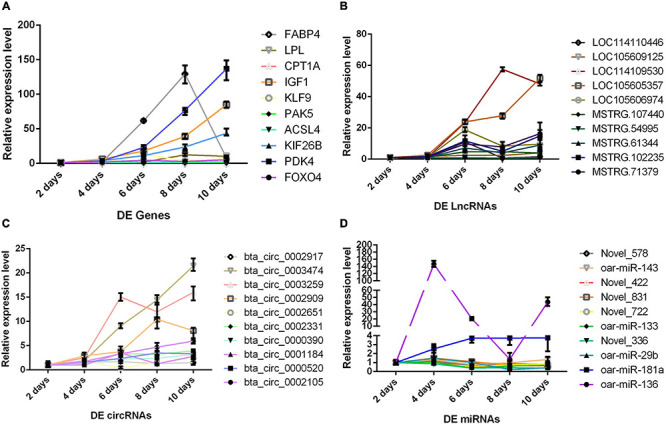
The change trend of differentially expressed RNA during adipocyte differentiation. The abscissa is the different days of cell culture, the ordinate is relative expression level, different colored lines represent different RNAs. **(A)** Messenger RNA (mRNA) results by qPCR. **(B)** Long non-coding RNA (lncRNA) results by qPCR. **(C)** Circular RNA (circRNA) results by qPCR. **(D)** MicroRNA (miRNA) results by qPCR.

Although we saw the expression alteration of the DERs between the P and M groups, it still cannot indicate that the RNAs were indeed involved in every period of adipocyte differentiation. We detected the expression trend of the DERs in the adipocyte differentiation period (2–10 days) by qPCR. The results showed that the RNAs had significant changes in the whole process of adipocyte differentiation (Figure 5). The RNAs also contained some pathways, such as miR-336/ACSL4/LncRNA-MSTRG71379/circRNA0002331, miR-422/FOXO4/LncRNA-MSTRG54995/circRNA0000520, miR-578/IGF1/LncRNA-MSTRG102235/circRNA0002971, and miR-722/PDK4/LncRNA-MSTRG107440/circRNA0002909. The DERs and networks were indeed involved in the process of sheep adipocyte differentiation. The core regulatory networks of four miRNAs are worthy of attention and in-depth studying.

## Discussion

Marbling is a vital standard to measure the quality of meat (Pethick et al., 2004). IMF content can significantly affect a marbling state. Increasing the accumulation of fat in intramuscular can promote the formation of meat marbling and improve meat properties such as taste, flavor, color, etc. (Dodson et al., 2010). The genetic factor leading to the speed of individual accumulating IMF content was different in the same breed and feeding conditions. The reasons may be which genetic factor determines the number of mammalian initial fat cells and the speed rate of adipocyte differentiation (Hocquette et al., 2010). Therefore, it is crucial to explore the potential molecular mechanisms of adipocyte differentiation. At present, a few studies provided candidate RNAs related to meat traits and were used for actual production (Hong et al., 2012; Yuan et al., 2013; Pertea et al., 2016). However, other non-coding RNAs and ceRNA remain less reported in sheep. In this study, we used high-throughput sequencing to compare RNA changes from preadipocytes to mature adipocytes. We obtained plenty of candidate genes and core regulatory networks affecting adipocyte differentiation by qPCR validation. These findings will enable us to understand the molecular mechanisms involved in adipocyte differentiation and expand new researching directions or biomarkers for improving the IMF and quality of mutton.

We found plenty of novel RNA transcripts in the study, especially non-coding RNAs. The number of novel miRNAs and lncRNAs dramatically surpassed that of the known ones. These results deserve attention. The main reasons may be that the reports on non-coding RNAs in sheep adipocyte differentiation were less and leading to lots of novel miRNAs and lncRNAs were not found. At the same time, there is lately no complete genome reference. In this study, a large number of genes involved in adipocyte differentiation were identified, especially well-known genes. The expression trend of the genes was consistent with what others reported (Prosdocimo et al., 2014; Olivecrona, 2016; Li et al., 2017; Kineman et al., 2018; Rodríguez-Calvo et al., 2019), such as LPL, FABP4, Forkhead box-O1 (FoxO1) (Chen et al., 2019), IGF1, and KLF family members. Some studies found that the selective knockdown of Matrix metalloproteinase 2 (MMP2) in mouse adipose cell lines led to a significant reduction (Bauters et al., 2015). Our sequencing data indicated that the MMP2 gene was also significantly increased in sheep adipocyte differentiation. Some reports suggested that Sirtuin 3 (SIRT3) promotes age-related adipogenesis and osteoclast production related to bone loss (Ho et al., 2017). The changing trend of the SIRT3 gene in these studies was consistent with our data. Reported miRNAs related to sheep lipid metabolism were less, especially only a few DEmiRNAs were found in the present study. miR-143 could be associated with obesity and could promote adipocyte differentiation in mice fed with a high-fat diet. We also found that miR-143 was significantly expressed during adipocyte differentiation (Bae et al., 2017). Some studies suggested that miR-181a promoted preadipocyte differentiation in porcine by directly targeting transforming growth factor-beta receptor 1 (TGFBR1) (Zhang et al., 2019). In this study, we also identified miR-181a and TGFBR1, and the expression trends were consistent with previous reports and findings. miR-27a can promote the proliferation of sheep preadipocytes and inhibit cell differentiation by regulating the expression of RXRα (Deng et al., 2020). Our experiment also identified the expression changes of miR-27a, but the trend was not statistically significant. One potential explanation for this is that our study missed the probability of the period of higher expression of miR-27a. To the best of our knowledge, only a limited number of reports have previously studied the expression profiles of lncRNAs and circRNAs concerning sheep lipid metabolism by searching the NCBI website. Although in the present study we found most lncRNAs and circRNAs affecting adipocyte differentiation, the DERs do not have too much literature for reference. The underlying function of DERs can provide a new research direction. In this study, some known genes affecting adipocyte differentiation had significant changes, such as PPARγ, CCAAT/enhancer-binding proteins (C/EBPα), and sterol regulatory element-binding protein 1 (SREBP1) (Sarjeant and Stephens, 2012). The main reasons may be what genes are only significantly expressed in the early stage of adipocyte differentiation. These trial samples were in preadipocyte and mature adipocyte stages, and the interval time is too long. Therefore, the experiment did not identify these well-known genes’ significant expression.

GO enrichment analysis showed that the results of four varieties of RNAs were similar; GO terms all enriched in cell part, cellular process, biological regulation, and binding. Non-coding RNAs made GO enrichment analysis based on targets or related to genes, so the final results were similar. KEGG pathway analysis results for the four varieties of RNAs showed that DERs mostly enriched in focal adhesion, PPAR signaling pathway, cancer, adipocytokine signaling pathway, MAPK signaling pathway, FoxO signal pathway, PI3K/Akt signaling pathways, and so on. A potential explanation for these findings could be that the non-coding RNAs may present different functions and spatiotemporal specific activities. However, we only studied the RNA expression in two periods of cell differentiation. Interestingly, we found many significantly dysregulated genes involved in cancer that participated in adipocyte differentiation.

We systematically constructed regulatory networks affecting adipocyte differentiation. The regulatory networks were complex; therefore, constructed various networks were necessary. In this study, IGF1 can participate in all regulatory networks, and it suggested that IGF1 played a vital role in adipocyte differentiation. We also noticed that miR-336, miR-422, miR-578, and miR-722 played core roles in all ceRNA regulatory networks. In this study, although the expression differences of the four new miRNAs (miR-336, miR-422, miR-578, and miR-722) did not seem to be significant in the qPCR results, we can see that the expressions of other network factors related to these four miRNAs are all significant, and these four genes also have significant changes at different stages of the adipocyte differentiation process. Interestingly, we found that all core miRNAs of the circRNA–miRNA–mRNA network participated in the four-element network. However, none of the core miRNAs of the lncRNA–miRNA–mRNA participated in the four-element network, and the reasons were unclear. We chose 40 DERs to detect the expression pattern in the process adipocyte differentiation, from which form many regulatory networks: miR-336/ACSL4/LncRNA-MSTRG71379/circRNA0002331, miR-422/FOXO4/LncRNA-MSTRG54995/circRNA0000520, miR-578/IGF1/LncRNA-MSTRG102235/circRNA0002971, miR-722/PDK4/LncRNA-MSTRG107440/circRNA0002909, and so on. The four networks and their core miRNAs were worthy of the next study. There were some problems encountered during the experiment. For example, the interval time from the P group to the M group was too long. Sequencing results may miss some highly expressed genes or non-coding RNAs in this period. We plan to add a new group of experiments between P and M groups to deal with the problems. In this study, trial data provided the DERs and pathways as practical value and reference for exploring the molecular mechanisms of adipocyte differentiation. This study not only adapts to livestock but also has valuable reference significance for human fat metabolism.

## Conclusion

We did whole-transcriptome sequencing in the sheep preadipocyte and the mature adipocyte group. We constructed for the first time comprehensive and systematic regulatory networks affecting sheep adipocyte differentiation. Sequencing data contained plenty of well-known and novel genes and non-coding RNAs; some of the DERs may be candidate RNAs, and they played a vital role in sheep adipocyte differentiation. The study identified that the four miRNAs (miR-336, miR-422, miR-578, and miR-722) were valuable for researching the molecular mechanisms of adipocyte differentiation. The four miRNAs involved in the networks also play crucial roles in the process and may be new biomarkers for improving IMF content and meat quality in sheep.

## Data Availability Statement

The datasets presented in this study can be found in online repositories. The names of the repository/repositories and accession number(s) are Sequence Read Archive—PRJNA639304, https://www.ncbi.nlm.nih.gov/search/all/?term=PRJNA639304.

## Ethics Statement

The animal study was reviewed and approved by AWEC 2019A05, 16 May 2019. Written informed consent was obtained from the owners for the participation of their animals in this study.

## Author Contributions

LZ and YC: conceptualization. YC: methodology and writing—review and editing. TW: software. CX: validation, formal analysis, writing—original draft preparation, and visualization. JL: investigation. HM: resources. LL: data curation. CW and ZY: supervision. HJ: project administration. HJ and HM: funding acquisition. All authors have read and agreed to the published version of the manuscript.

## Conflict of Interest

The authors declare that the research was conducted in the absence of any commercial or financial relationships that could be construed as a potential conflict of interest.

## Publisher’s Note

All claims expressed in this article are solely those of the authors and do not necessarily represent those of their affiliated organizations, or those of the publisher, the editors and the reviewers. Any product that may be evaluated in this article, or claim that may be made by its manufacturer, is not guaranteed or endorsed by the publisher.

## Footnotes

1
http://ccb.jhu.edu/software/stringtie/gff.shtml

2
https://submit.ncbi.nlm.nih.gov/subs/sra/SUB7601005/overview

3
https://submit.ncbi.nlm.nih.gov/subs/sra/SUB7600133/overview

## Supplementary Material

Supplementary Figure 1 Volcano map of different groups of differential RNAs. **(A–C)** Expression profiles of mRNAs and LncRNAs (M1 vs. P1, M2 vs. P2, M3 vs. P3). **(D–F)** Expression profiles of circRNAs (M1 vs. P1, M2 vs. P2, M3 vs. P3). Green points represent down-regulated RNAs; red points represent up-regulated RNAs; gray points represent not significantly expressed RNAs in the volcano plots. **(G–I)** Expression profiles of miRNAs (M1 vs. P1, M2 vs. P2, M3 vs. P3). Blue points represent down-regulated RNAs; yellow points represent up-regulated RNAs; gray points represent not significantly expressed RNAs in the volcano plots. X-axis: Fold change log2 ratio of RNAs. Y-axis: false discovery rate values (-log10 transformed).

Supplementary Figure 2 Cluster map of different groups of differential RNAs. **(A)** Cluster analysis of mRNAs. **(B)** Cluster analysis of LncRNAs. **(C)** Cluster analysis of circRNAs. **(D)** Cluster analysis of miRNAs. The yellow and blue regions represent increased and decreased RNAs.

Supplementary Figure 3 Gene ontology (GO) analysis of different groups of differential RNAs. **(A)** GO annotations of DE mRNAs. **(B)** GO annotations of DE LncRNAs. **(C)** GO annotations of DE circRNAs. **(D)** GO annotations of DE miRNAs.

Supplementary Figure 4 KEGG pathway analysis of different groups of differential RNAs. **(A)** KEGG pathway of DE mRNAs. **(B)** KEGG pathway of DE LncRNAs. **(C)** KEGG pathway of DE circRNAs. **(D)** KEGG pathway of DE miRNAs.

Supplementary Table 1 Primer sequences of mRNAs.

Supplementary Table 2 Primer sequences of LncRNAs.

Supplementary Table 3 Primer sequences of circRNAs.

Supplementary Table 4 Primer sequences of miRNAs.

Supplementary Table 5 List of 1,196 DEmRNAs.

Supplementary Table 6 List of 754 DELncRNAs.

Supplementary Table 7 List of 17 DEcircRNAs.

Supplementary Table 8 List of 100 DEmiRNAs.

Supplementary Table 9 Data of LncRNA binds to miRNA.

Supplementary Table 10 Data of LncRNA binds to mRNA.

Supplementary Table 11 Data of circRNA absorbs miRNA.

Supplementary Table 12 Data of miRNA bind to mRNA.

Supplementary Table 13 LncRNA-mRNA-miRNA correlation data.

Supplementary Table 14 circRNA-mRNA-miRNA correlation data.

Supplementary Table 15 circRNA-LncRNA-mRNA-miRNA correlation data.
