# Supplementary figures and images for: Whole-Transcriptome Analysis of Preadipocyte and Adipocyte and Construction of Regulatory Networks to Investigate Lipid Metabolism in Sheep
Source: Front Genet. 2021 Jul 29;12:662143. doi: 10.3389/fgene.2021.662143 (PMC8358208; doi:10.3389/fgene.2021.662143)

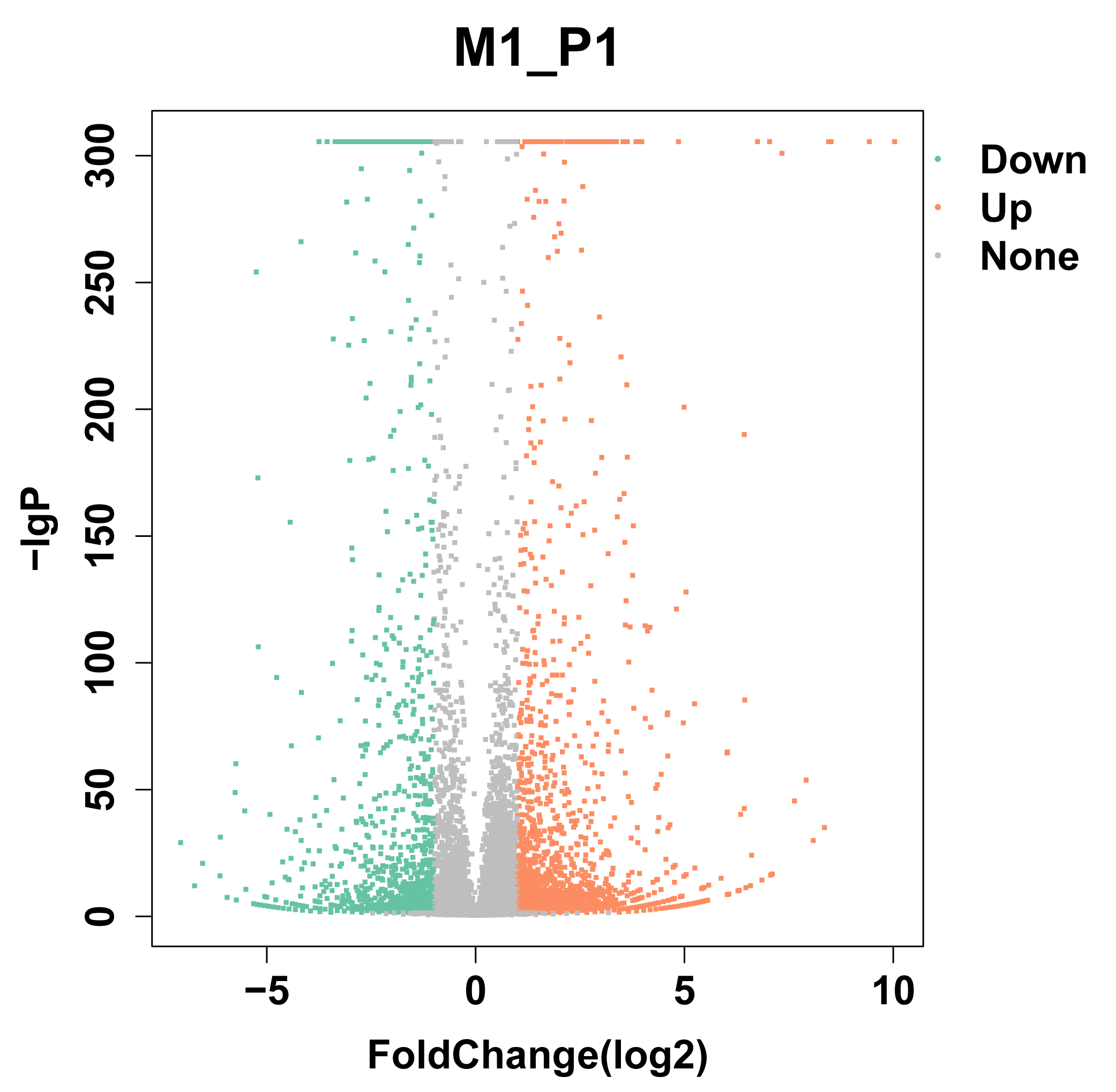

Supplement: Supplementary Figure 1 — Volcano map of different groups of differential RNAs. (A–C) Expression profiles of mRNAs and LncRNAs (M1 vs. P1, M2 vs. P2, M3 vs. P3). (D–F) Expression profiles of circRNAs (M1 vs. P1, M2 vs. P2, M3 vs. P3). Green points represent down-regulated RNAs; red points represent up-regulated RNAs; gray points represent not significantly expressed RNAs in the volcano plots. (G–I) Expression profiles of miRNAs (M1 vs. P1, M2 vs. P2, M3 vs. P3). Blue points represent down-regulated RNAs; yellow points represent up-regulated RNAs; gray points represent not significantly expressed RNAs in the volcano plots. X-axis: Fold change log2 ratio of RNAs. Y-axis: false discovery rate values (-log10 transformed). [file Data_Sheet_1.ZIP › Supplementary figures and tables/FigureS1A.tif]

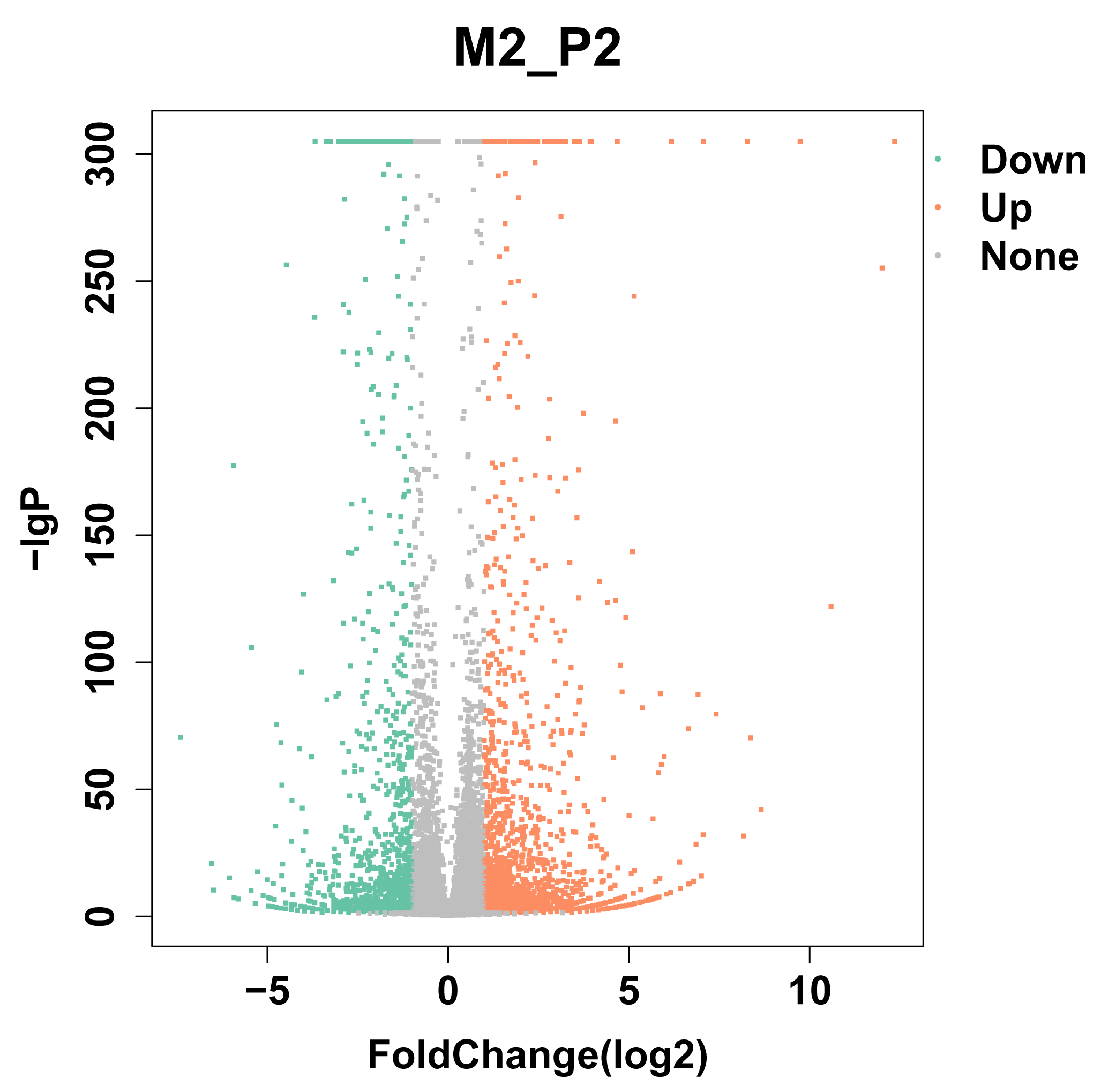

Supplement: Supplementary Figure 1 — Volcano map of different groups of differential RNAs. (A–C) Expression profiles of mRNAs and LncRNAs (M1 vs. P1, M2 vs. P2, M3 vs. P3). (D–F) Expression profiles of circRNAs (M1 vs. P1, M2 vs. P2, M3 vs. P3). Green points represent down-regulated RNAs; red points represent up-regulated RNAs; gray points represent not significantly expressed RNAs in the volcano plots. (G–I) Expression profiles of miRNAs (M1 vs. P1, M2 vs. P2, M3 vs. P3). Blue points represent down-regulated RNAs; yellow points represent up-regulated RNAs; gray points represent not significantly expressed RNAs in the volcano plots. X-axis: Fold change log2 ratio of RNAs. Y-axis: false discovery rate values (-log10 transformed). [file Data_Sheet_1.ZIP › Supplementary figures and tables/FigureS1B.tif]

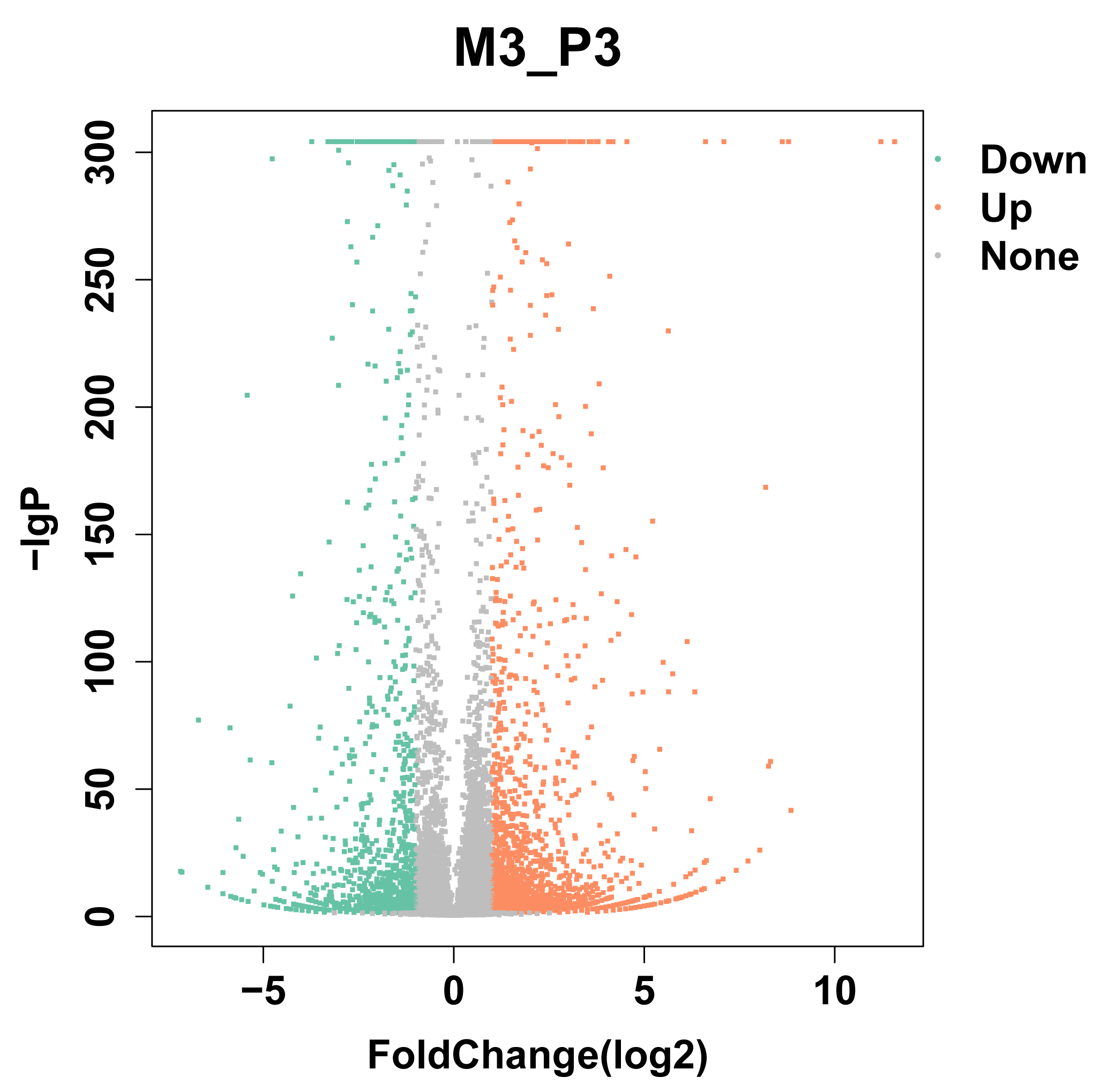

Supplement: Supplementary Figure 1 — Volcano map of different groups of differential RNAs. (A–C) Expression profiles of mRNAs and LncRNAs (M1 vs. P1, M2 vs. P2, M3 vs. P3). (D–F) Expression profiles of circRNAs (M1 vs. P1, M2 vs. P2, M3 vs. P3). Green points represent down-regulated RNAs; red points represent up-regulated RNAs; gray points represent not significantly expressed RNAs in the volcano plots. (G–I) Expression profiles of miRNAs (M1 vs. P1, M2 vs. P2, M3 vs. P3). Blue points represent down-regulated RNAs; yellow points represent up-regulated RNAs; gray points represent not significantly expressed RNAs in the volcano plots. X-axis: Fold change log2 ratio of RNAs. Y-axis: false discovery rate values (-log10 transformed). [file Data_Sheet_1.ZIP › Supplementary figures and tables/FigureS1C.tif]

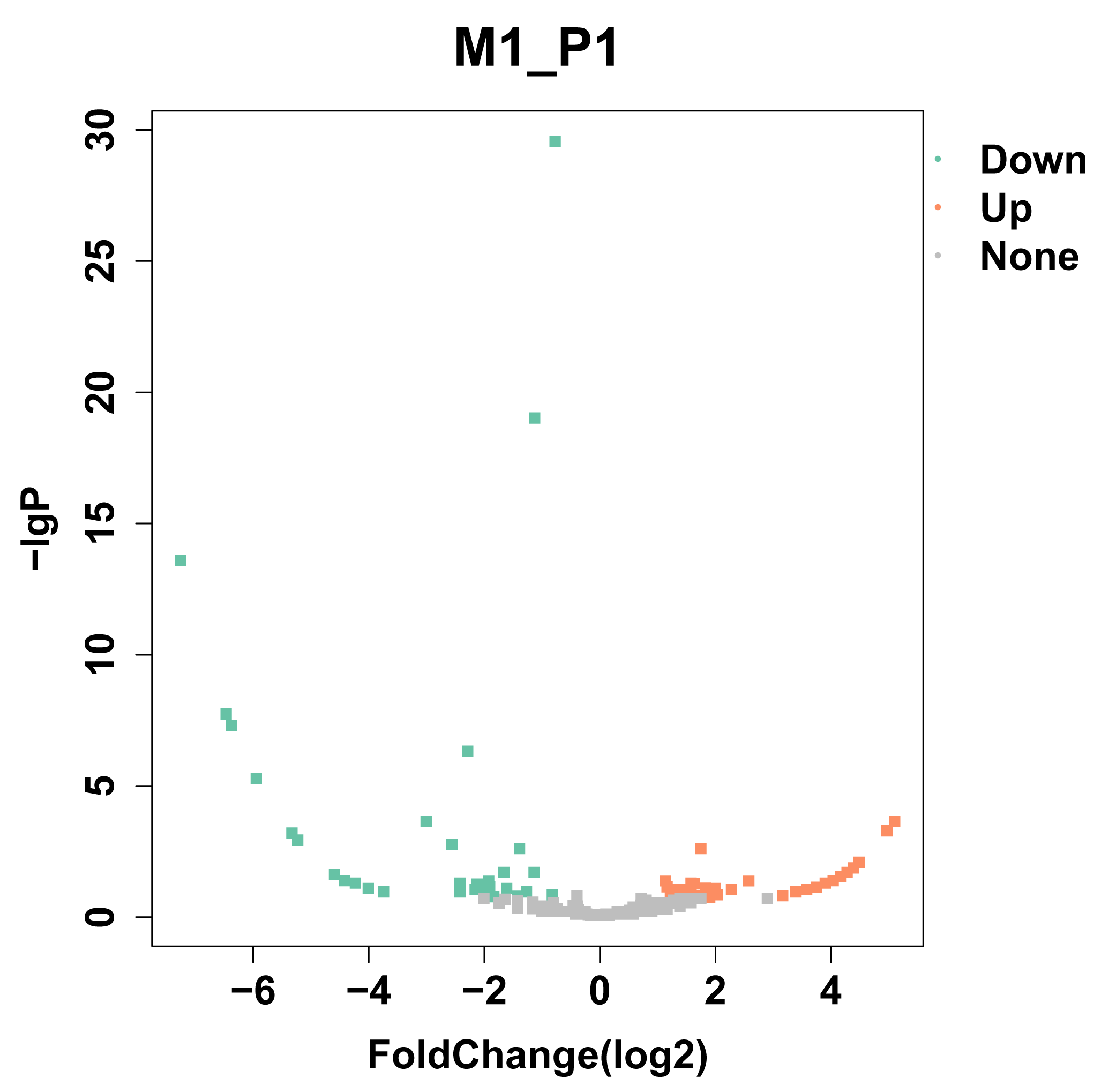

Supplement: Supplementary Figure 1 — Volcano map of different groups of differential RNAs. (A–C) Expression profiles of mRNAs and LncRNAs (M1 vs. P1, M2 vs. P2, M3 vs. P3). (D–F) Expression profiles of circRNAs (M1 vs. P1, M2 vs. P2, M3 vs. P3). Green points represent down-regulated RNAs; red points represent up-regulated RNAs; gray points represent not significantly expressed RNAs in the volcano plots. (G–I) Expression profiles of miRNAs (M1 vs. P1, M2 vs. P2, M3 vs. P3). Blue points represent down-regulated RNAs; yellow points represent up-regulated RNAs; gray points represent not significantly expressed RNAs in the volcano plots. X-axis: Fold change log2 ratio of RNAs. Y-axis: false discovery rate values (-log10 transformed). [file Data_Sheet_1.ZIP › Supplementary figures and tables/FigureS1D.tif]

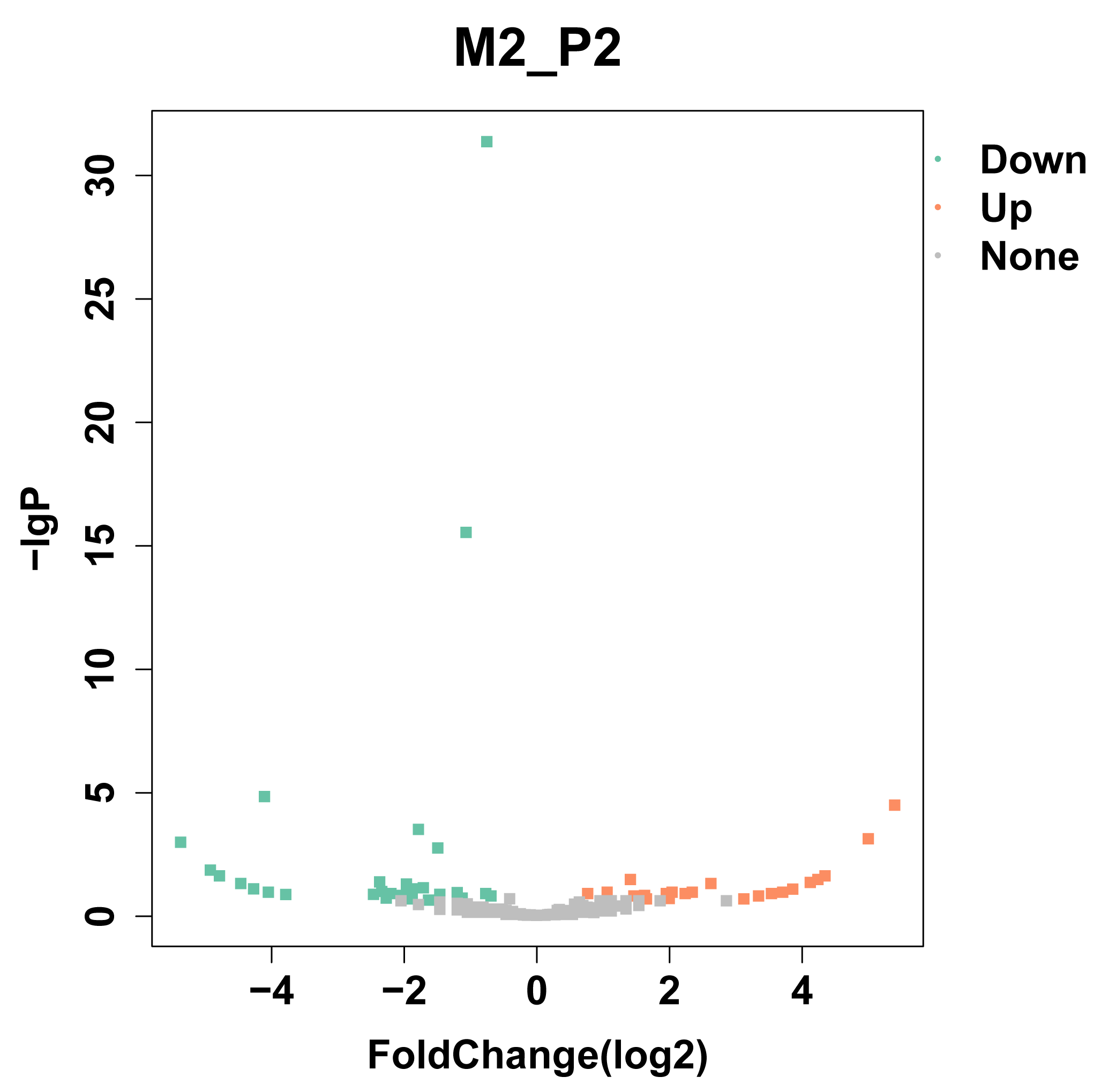

Supplement: Supplementary Figure 1 — Volcano map of different groups of differential RNAs. (A–C) Expression profiles of mRNAs and LncRNAs (M1 vs. P1, M2 vs. P2, M3 vs. P3). (D–F) Expression profiles of circRNAs (M1 vs. P1, M2 vs. P2, M3 vs. P3). Green points represent down-regulated RNAs; red points represent up-regulated RNAs; gray points represent not significantly expressed RNAs in the volcano plots. (G–I) Expression profiles of miRNAs (M1 vs. P1, M2 vs. P2, M3 vs. P3). Blue points represent down-regulated RNAs; yellow points represent up-regulated RNAs; gray points represent not significantly expressed RNAs in the volcano plots. X-axis: Fold change log2 ratio of RNAs. Y-axis: false discovery rate values (-log10 transformed). [file Data_Sheet_1.ZIP › Supplementary figures and tables/FigureS1E.tif]

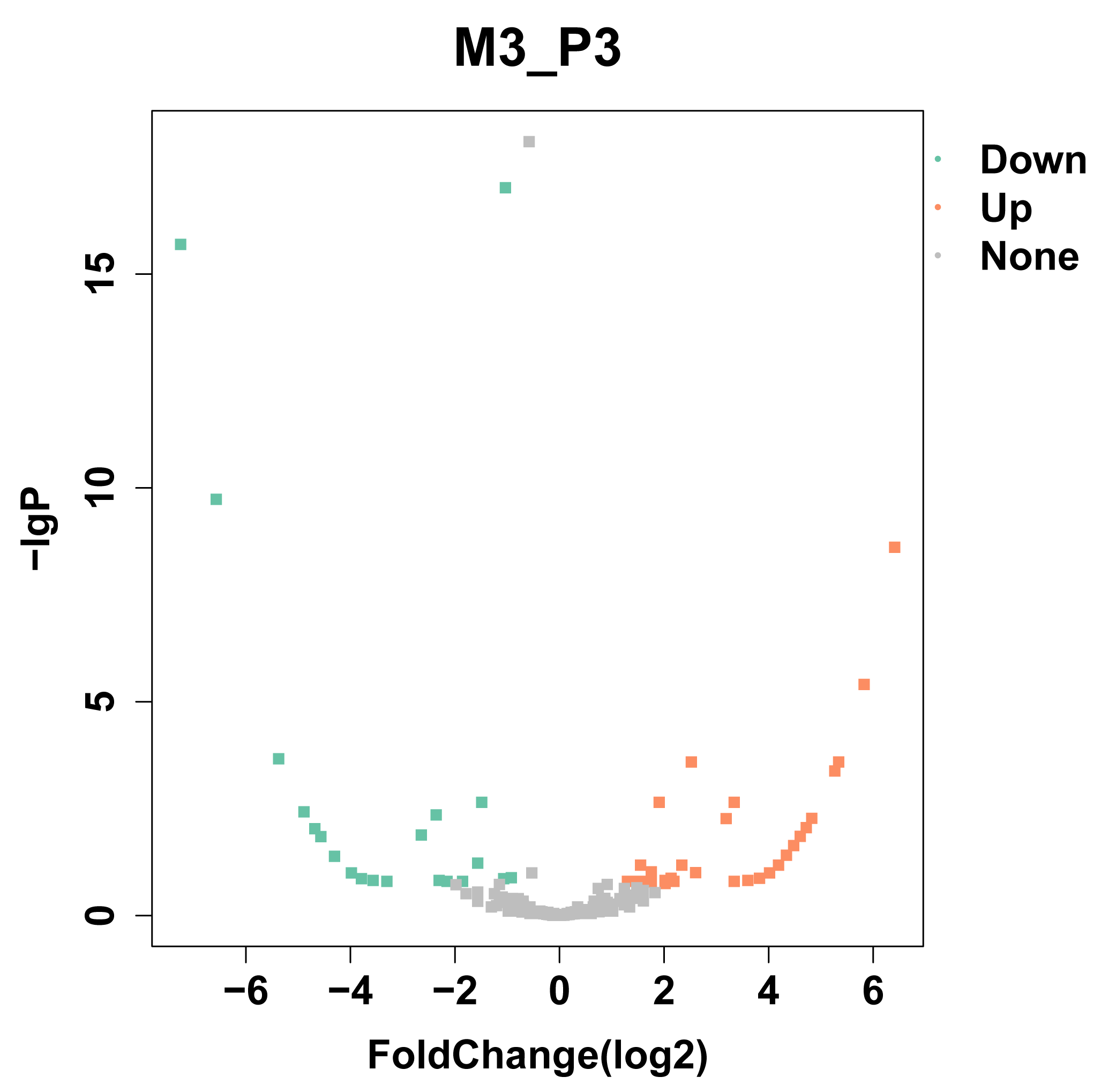

Supplement: Supplementary Figure 1 — Volcano map of different groups of differential RNAs. (A–C) Expression profiles of mRNAs and LncRNAs (M1 vs. P1, M2 vs. P2, M3 vs. P3). (D–F) Expression profiles of circRNAs (M1 vs. P1, M2 vs. P2, M3 vs. P3). Green points represent down-regulated RNAs; red points represent up-regulated RNAs; gray points represent not significantly expressed RNAs in the volcano plots. (G–I) Expression profiles of miRNAs (M1 vs. P1, M2 vs. P2, M3 vs. P3). Blue points represent down-regulated RNAs; yellow points represent up-regulated RNAs; gray points represent not significantly expressed RNAs in the volcano plots. X-axis: Fold change log2 ratio of RNAs. Y-axis: false discovery rate values (-log10 transformed). [file Data_Sheet_1.ZIP › Supplementary figures and tables/FigureS1F.tif]

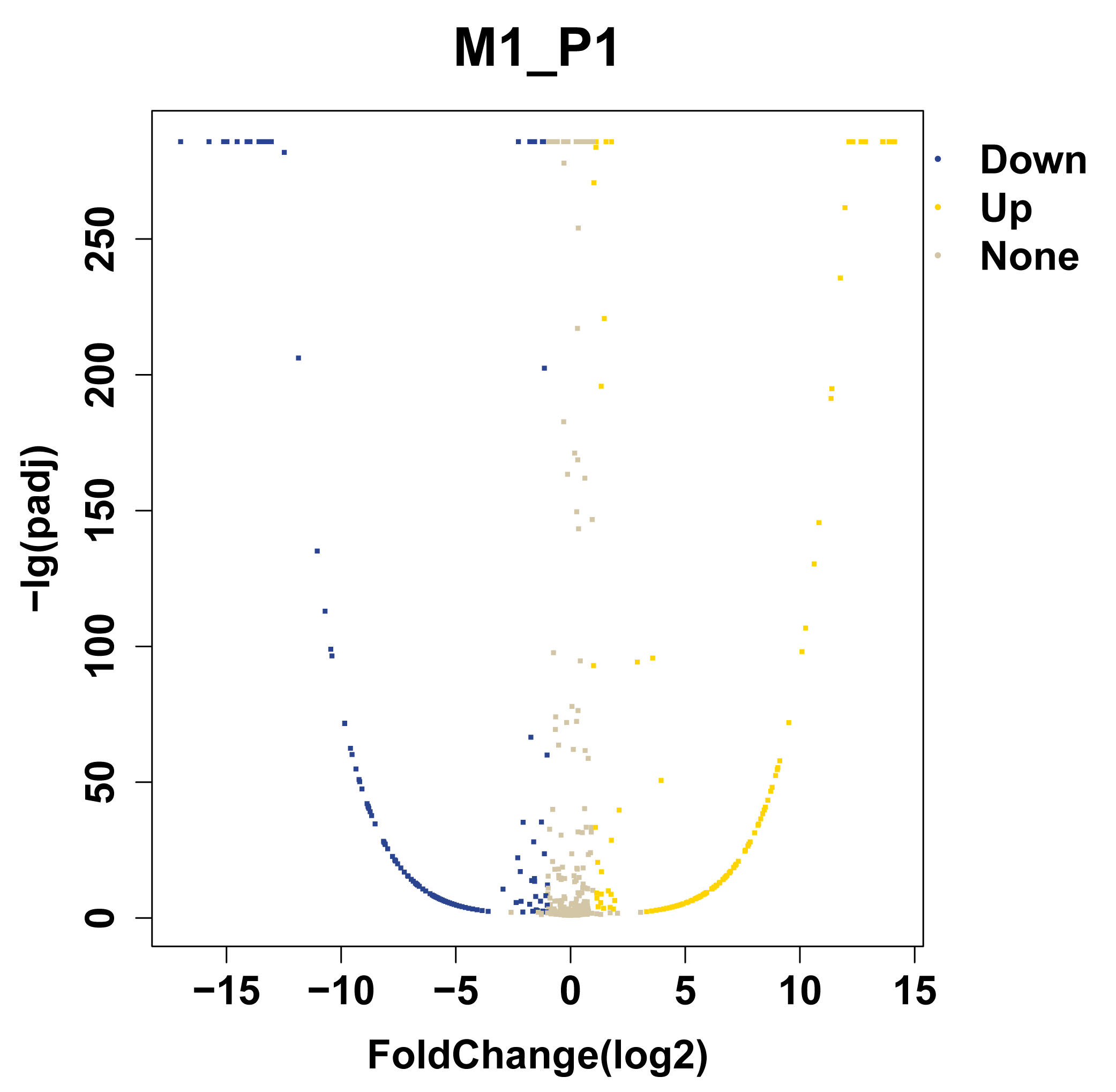

Supplement: Supplementary Figure 1 — Volcano map of different groups of differential RNAs. (A–C) Expression profiles of mRNAs and LncRNAs (M1 vs. P1, M2 vs. P2, M3 vs. P3). (D–F) Expression profiles of circRNAs (M1 vs. P1, M2 vs. P2, M3 vs. P3). Green points represent down-regulated RNAs; red points represent up-regulated RNAs; gray points represent not significantly expressed RNAs in the volcano plots. (G–I) Expression profiles of miRNAs (M1 vs. P1, M2 vs. P2, M3 vs. P3). Blue points represent down-regulated RNAs; yellow points represent up-regulated RNAs; gray points represent not significantly expressed RNAs in the volcano plots. X-axis: Fold change log2 ratio of RNAs. Y-axis: false discovery rate values (-log10 transformed). [file Data_Sheet_1.ZIP › Supplementary figures and tables/FigureS1G.tif]

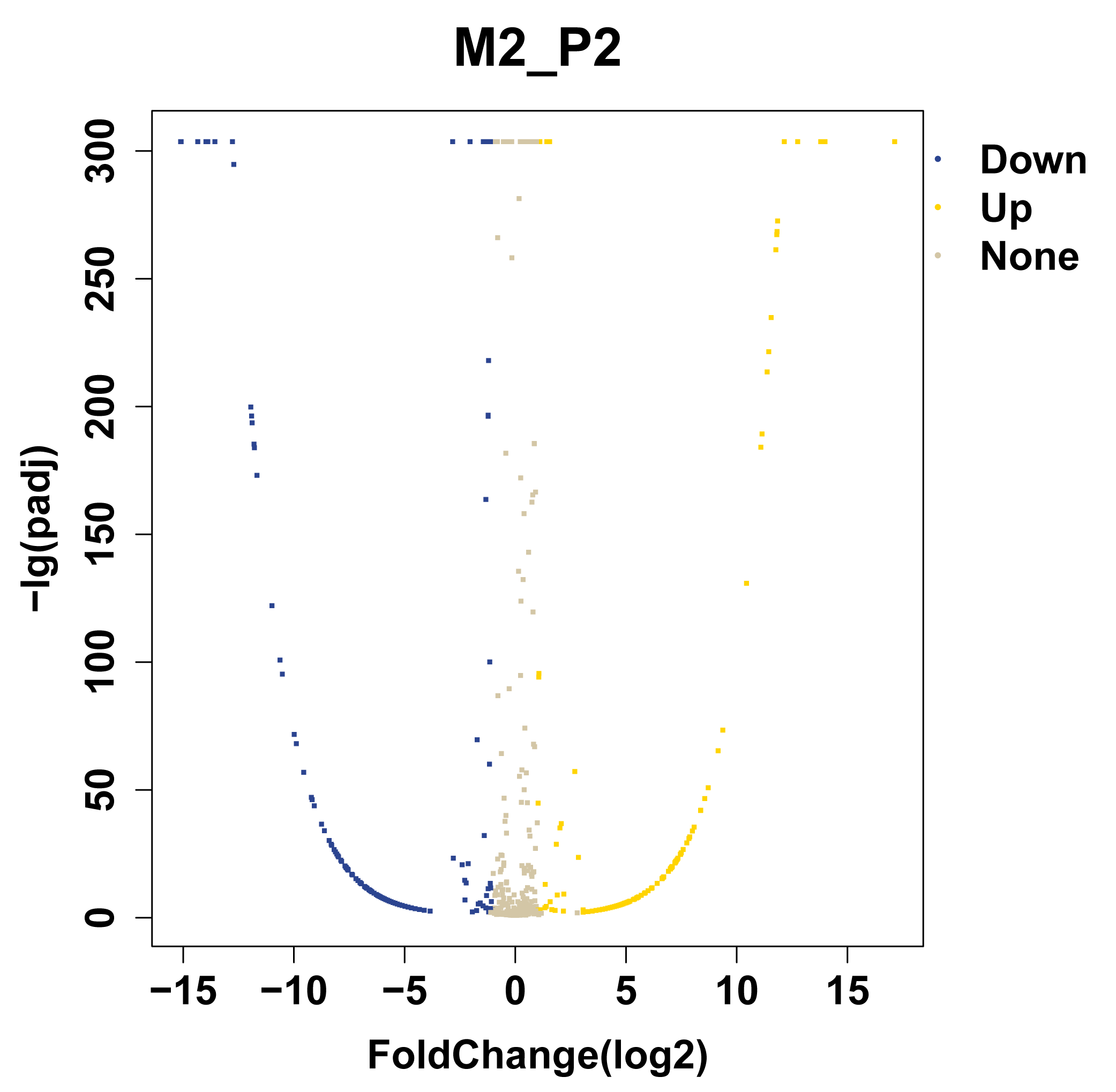

Supplement: Supplementary Figure 1 — Volcano map of different groups of differential RNAs. (A–C) Expression profiles of mRNAs and LncRNAs (M1 vs. P1, M2 vs. P2, M3 vs. P3). (D–F) Expression profiles of circRNAs (M1 vs. P1, M2 vs. P2, M3 vs. P3). Green points represent down-regulated RNAs; red points represent up-regulated RNAs; gray points represent not significantly expressed RNAs in the volcano plots. (G–I) Expression profiles of miRNAs (M1 vs. P1, M2 vs. P2, M3 vs. P3). Blue points represent down-regulated RNAs; yellow points represent up-regulated RNAs; gray points represent not significantly expressed RNAs in the volcano plots. X-axis: Fold change log2 ratio of RNAs. Y-axis: false discovery rate values (-log10 transformed). [file Data_Sheet_1.ZIP › Supplementary figures and tables/FigureS1H.tif]

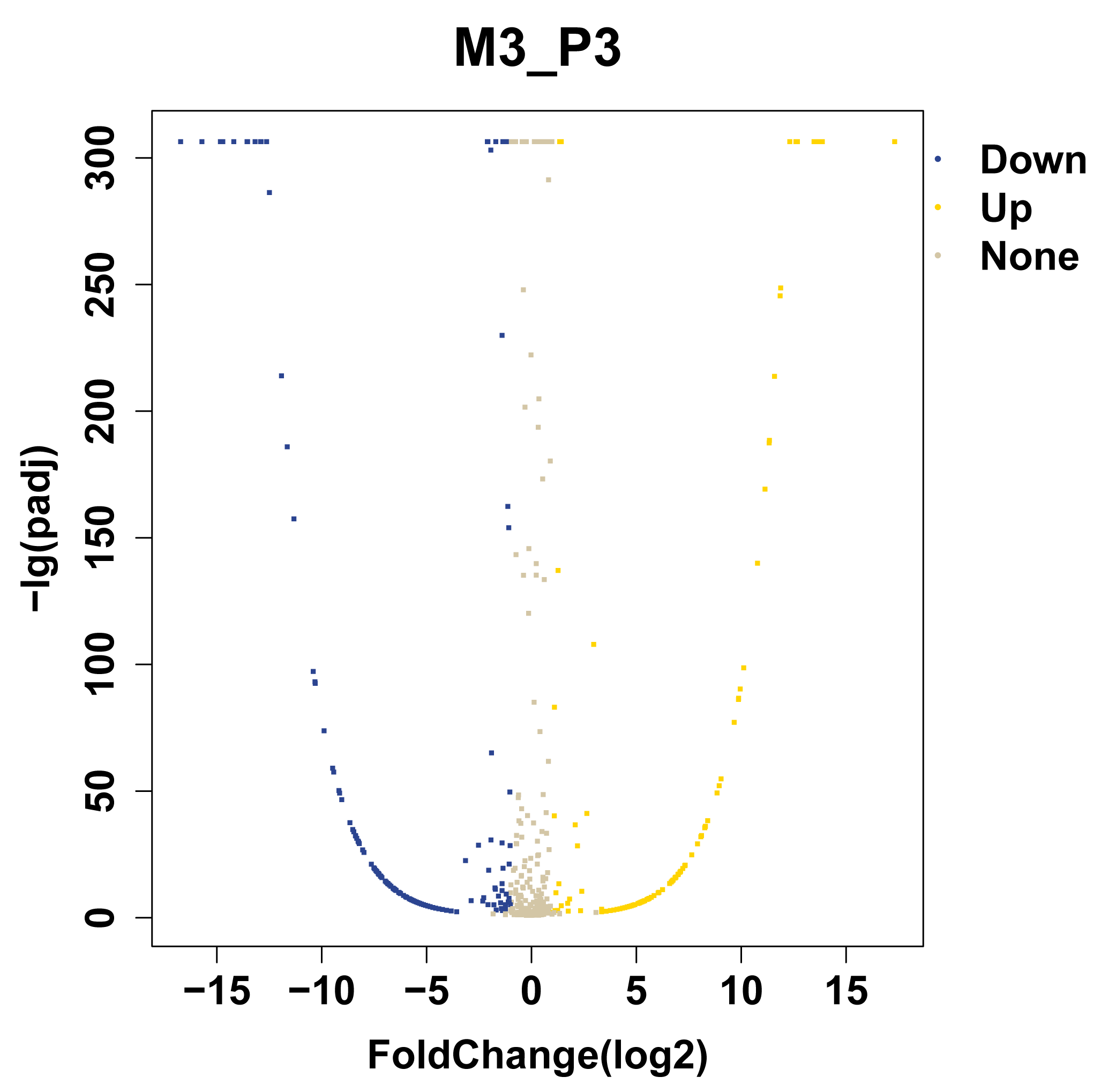

Supplement: Supplementary Figure 1 — Volcano map of different groups of differential RNAs. (A–C) Expression profiles of mRNAs and LncRNAs (M1 vs. P1, M2 vs. P2, M3 vs. P3). (D–F) Expression profiles of circRNAs (M1 vs. P1, M2 vs. P2, M3 vs. P3). Green points represent down-regulated RNAs; red points represent up-regulated RNAs; gray points represent not significantly expressed RNAs in the volcano plots. (G–I) Expression profiles of miRNAs (M1 vs. P1, M2 vs. P2, M3 vs. P3). Blue points represent down-regulated RNAs; yellow points represent up-regulated RNAs; gray points represent not significantly expressed RNAs in the volcano plots. X-axis: Fold change log2 ratio of RNAs. Y-axis: false discovery rate values (-log10 transformed). [file Data_Sheet_1.ZIP › Supplementary figures and tables/FigureS1I.tif]

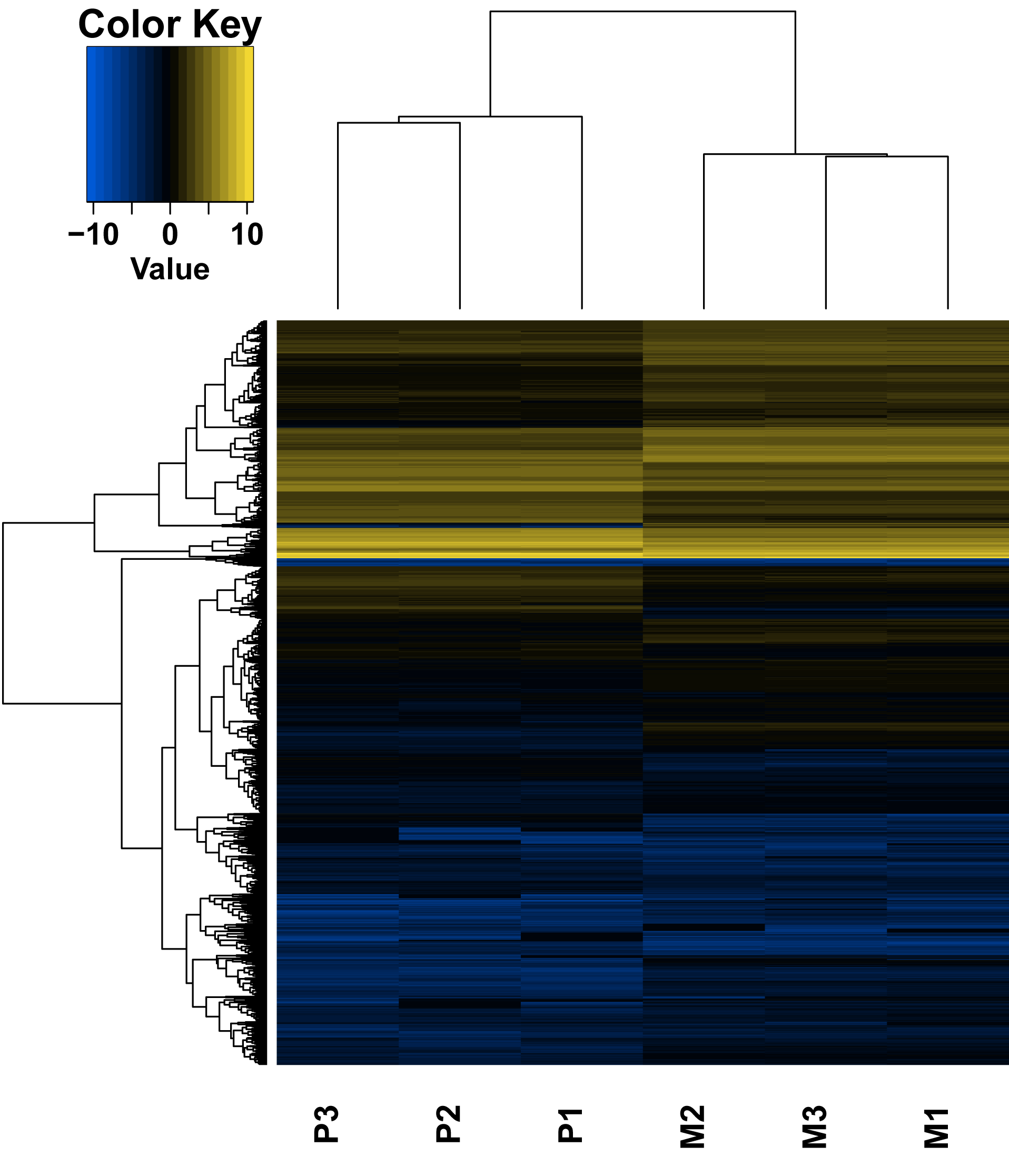

Supplement: Supplementary Figure 1 — Volcano map of different groups of differential RNAs. (A–C) Expression profiles of mRNAs and LncRNAs (M1 vs. P1, M2 vs. P2, M3 vs. P3). (D–F) Expression profiles of circRNAs (M1 vs. P1, M2 vs. P2, M3 vs. P3). Green points represent down-regulated RNAs; red points represent up-regulated RNAs; gray points represent not significantly expressed RNAs in the volcano plots. (G–I) Expression profiles of miRNAs (M1 vs. P1, M2 vs. P2, M3 vs. P3). Blue points represent down-regulated RNAs; yellow points represent up-regulated RNAs; gray points represent not significantly expressed RNAs in the volcano plots. X-axis: Fold change log2 ratio of RNAs. Y-axis: false discovery rate values (-log10 transformed). [file Data_Sheet_1.ZIP › Supplementary figures and tables/FigureS2A.tif]

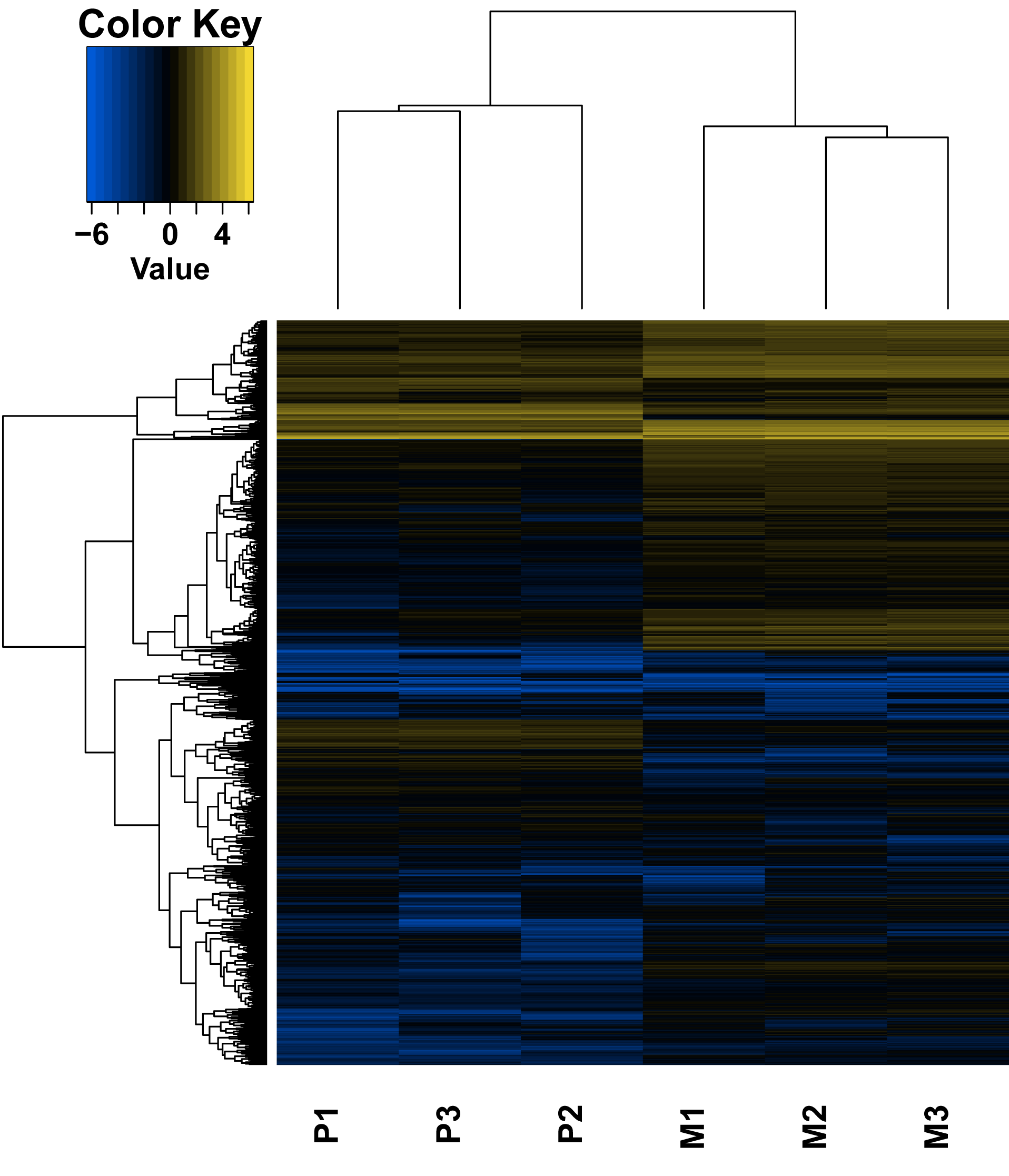

Supplement: Supplementary Figure 1 — Volcano map of different groups of differential RNAs. (A–C) Expression profiles of mRNAs and LncRNAs (M1 vs. P1, M2 vs. P2, M3 vs. P3). (D–F) Expression profiles of circRNAs (M1 vs. P1, M2 vs. P2, M3 vs. P3). Green points represent down-regulated RNAs; red points represent up-regulated RNAs; gray points represent not significantly expressed RNAs in the volcano plots. (G–I) Expression profiles of miRNAs (M1 vs. P1, M2 vs. P2, M3 vs. P3). Blue points represent down-regulated RNAs; yellow points represent up-regulated RNAs; gray points represent not significantly expressed RNAs in the volcano plots. X-axis: Fold change log2 ratio of RNAs. Y-axis: false discovery rate values (-log10 transformed). [file Data_Sheet_1.ZIP › Supplementary figures and tables/FigureS2B.tif]

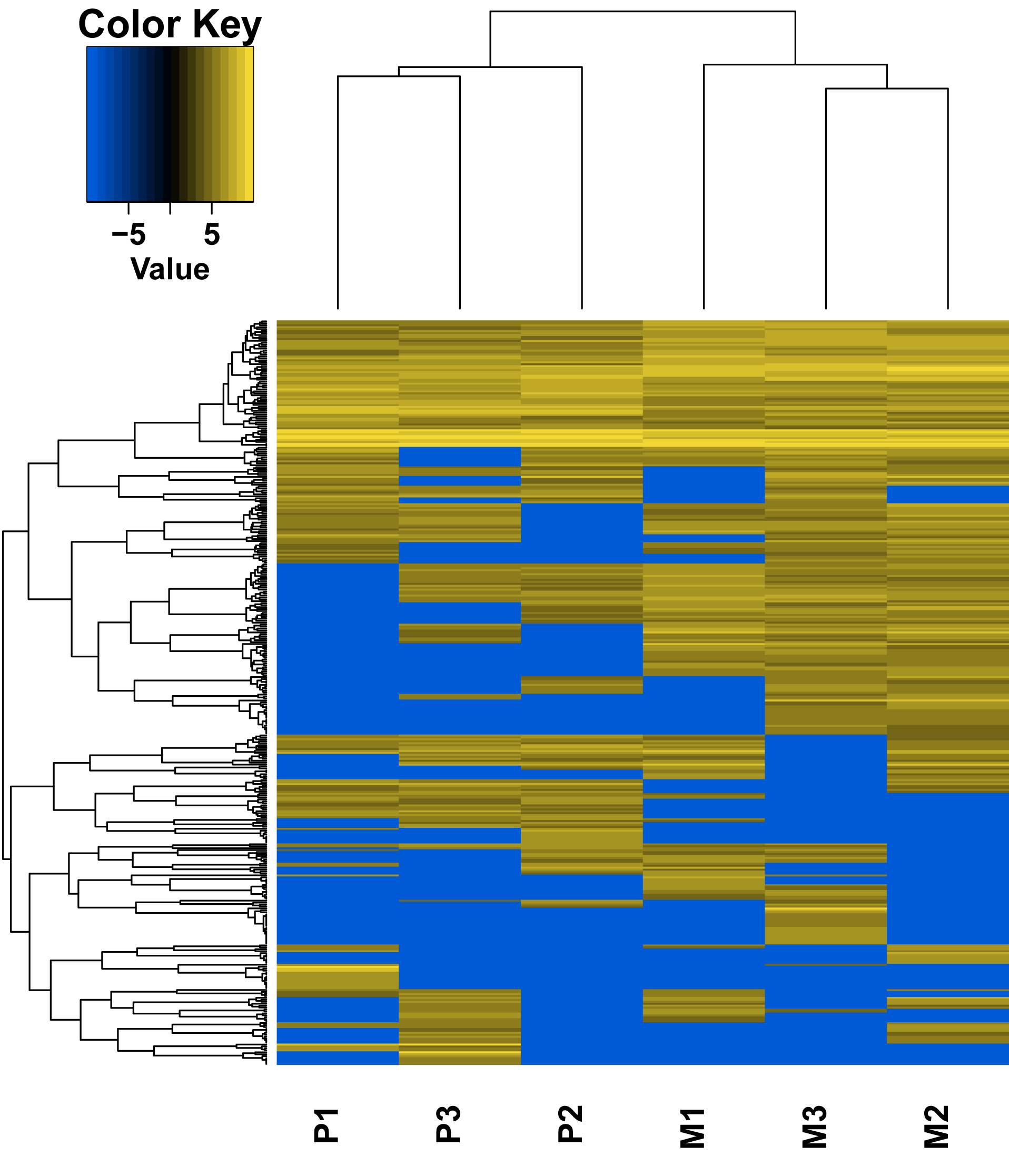

Supplement: Supplementary Figure 1 — Volcano map of different groups of differential RNAs. (A–C) Expression profiles of mRNAs and LncRNAs (M1 vs. P1, M2 vs. P2, M3 vs. P3). (D–F) Expression profiles of circRNAs (M1 vs. P1, M2 vs. P2, M3 vs. P3). Green points represent down-regulated RNAs; red points represent up-regulated RNAs; gray points represent not significantly expressed RNAs in the volcano plots. (G–I) Expression profiles of miRNAs (M1 vs. P1, M2 vs. P2, M3 vs. P3). Blue points represent down-regulated RNAs; yellow points represent up-regulated RNAs; gray points represent not significantly expressed RNAs in the volcano plots. X-axis: Fold change log2 ratio of RNAs. Y-axis: false discovery rate values (-log10 transformed). [file Data_Sheet_1.ZIP › Supplementary figures and tables/FigureS2C.tif]

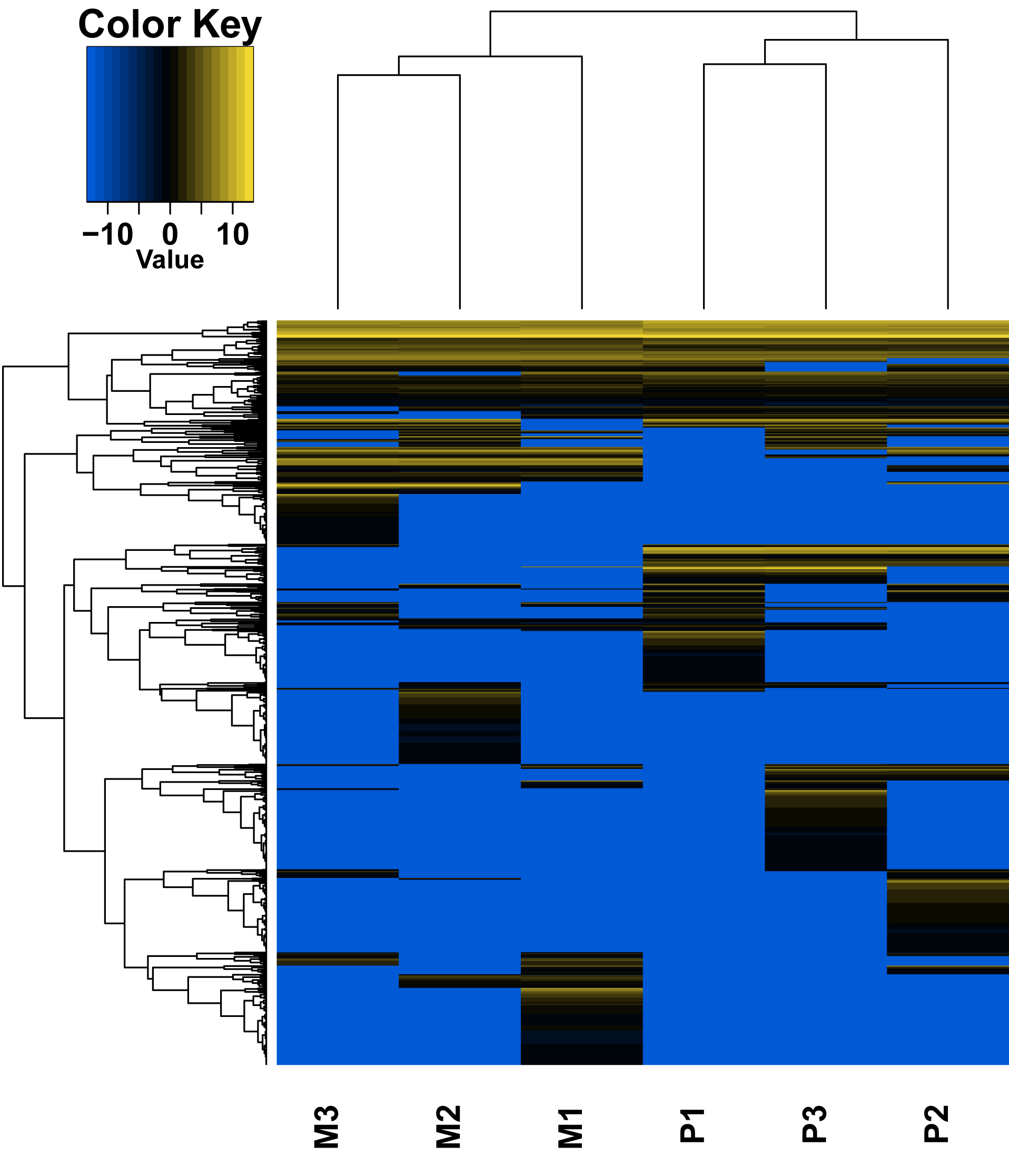

Supplement: Supplementary Figure 1 — Volcano map of different groups of differential RNAs. (A–C) Expression profiles of mRNAs and LncRNAs (M1 vs. P1, M2 vs. P2, M3 vs. P3). (D–F) Expression profiles of circRNAs (M1 vs. P1, M2 vs. P2, M3 vs. P3). Green points represent down-regulated RNAs; red points represent up-regulated RNAs; gray points represent not significantly expressed RNAs in the volcano plots. (G–I) Expression profiles of miRNAs (M1 vs. P1, M2 vs. P2, M3 vs. P3). Blue points represent down-regulated RNAs; yellow points represent up-regulated RNAs; gray points represent not significantly expressed RNAs in the volcano plots. X-axis: Fold change log2 ratio of RNAs. Y-axis: false discovery rate values (-log10 transformed). [file Data_Sheet_1.ZIP › Supplementary figures and tables/FigureS2D.tif]

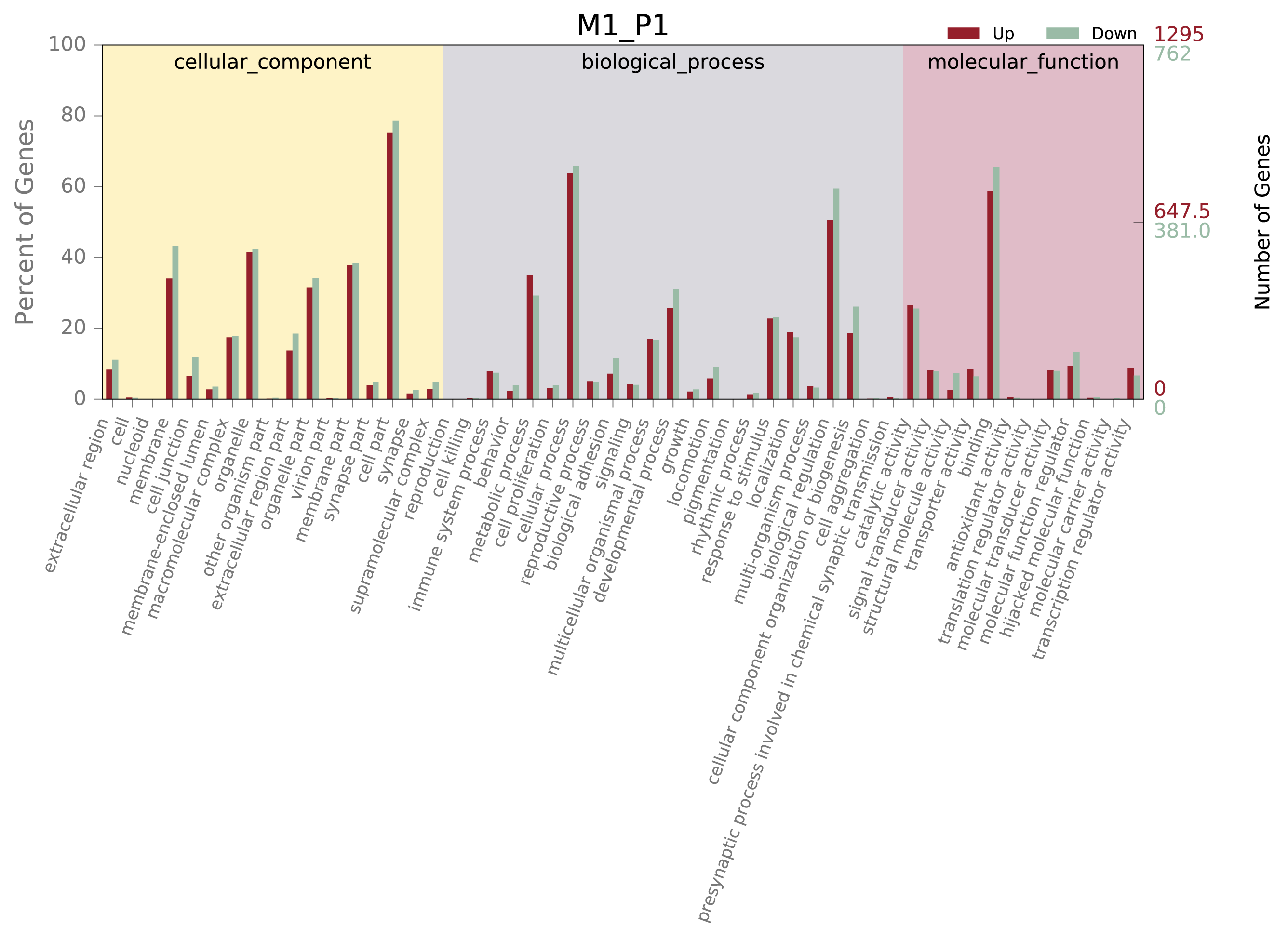

Supplement: Supplementary Figure 1 — Volcano map of different groups of differential RNAs. (A–C) Expression profiles of mRNAs and LncRNAs (M1 vs. P1, M2 vs. P2, M3 vs. P3). (D–F) Expression profiles of circRNAs (M1 vs. P1, M2 vs. P2, M3 vs. P3). Green points represent down-regulated RNAs; red points represent up-regulated RNAs; gray points represent not significantly expressed RNAs in the volcano plots. (G–I) Expression profiles of miRNAs (M1 vs. P1, M2 vs. P2, M3 vs. P3). Blue points represent down-regulated RNAs; yellow points represent up-regulated RNAs; gray points represent not significantly expressed RNAs in the volcano plots. X-axis: Fold change log2 ratio of RNAs. Y-axis: false discovery rate values (-log10 transformed). [file Data_Sheet_1.ZIP › Supplementary figures and tables/FigureS3A.tif]

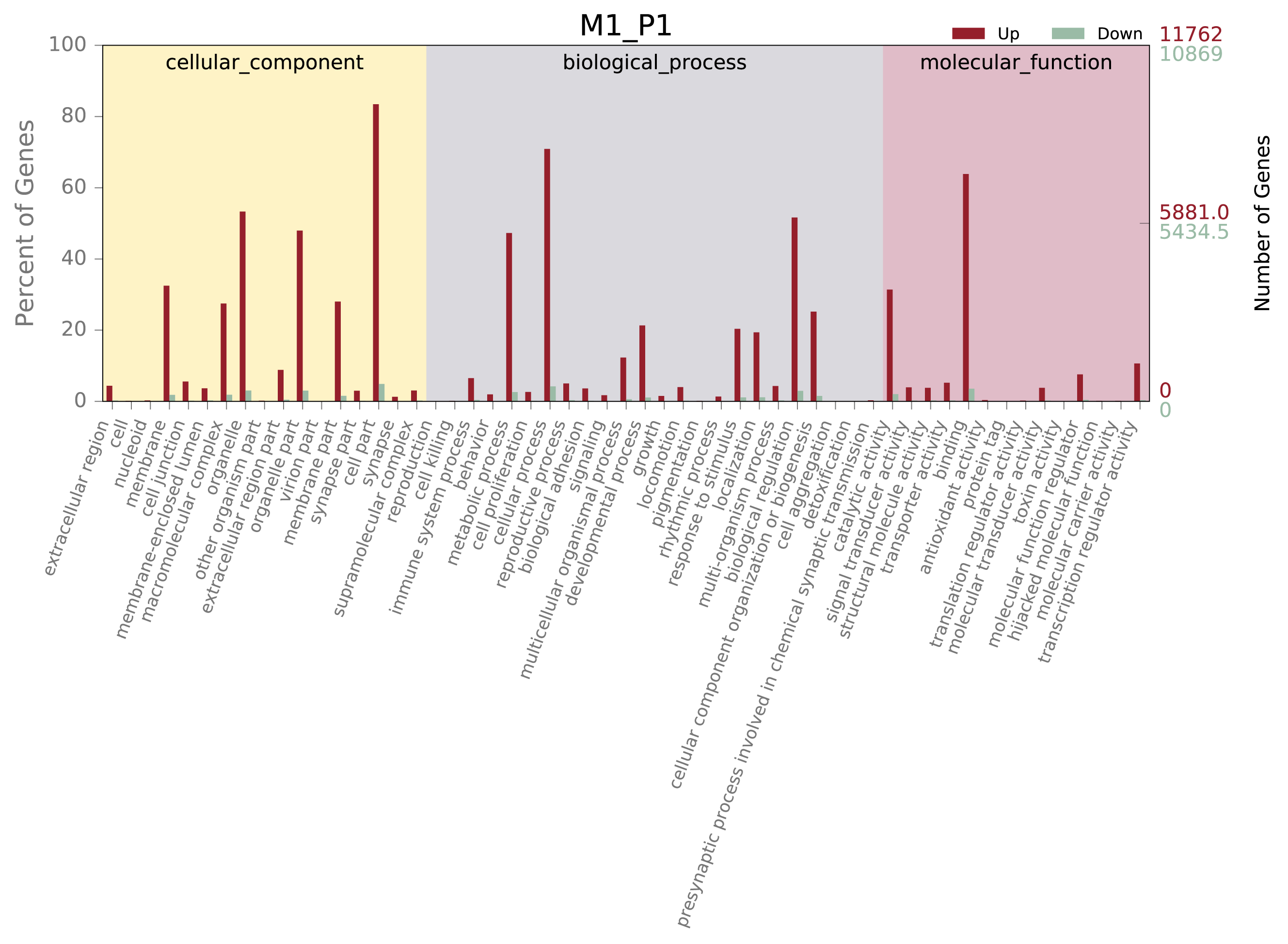

Supplement: Supplementary Figure 1 — Volcano map of different groups of differential RNAs. (A–C) Expression profiles of mRNAs and LncRNAs (M1 vs. P1, M2 vs. P2, M3 vs. P3). (D–F) Expression profiles of circRNAs (M1 vs. P1, M2 vs. P2, M3 vs. P3). Green points represent down-regulated RNAs; red points represent up-regulated RNAs; gray points represent not significantly expressed RNAs in the volcano plots. (G–I) Expression profiles of miRNAs (M1 vs. P1, M2 vs. P2, M3 vs. P3). Blue points represent down-regulated RNAs; yellow points represent up-regulated RNAs; gray points represent not significantly expressed RNAs in the volcano plots. X-axis: Fold change log2 ratio of RNAs. Y-axis: false discovery rate values (-log10 transformed). [file Data_Sheet_1.ZIP › Supplementary figures and tables/FigureS3B.tif]

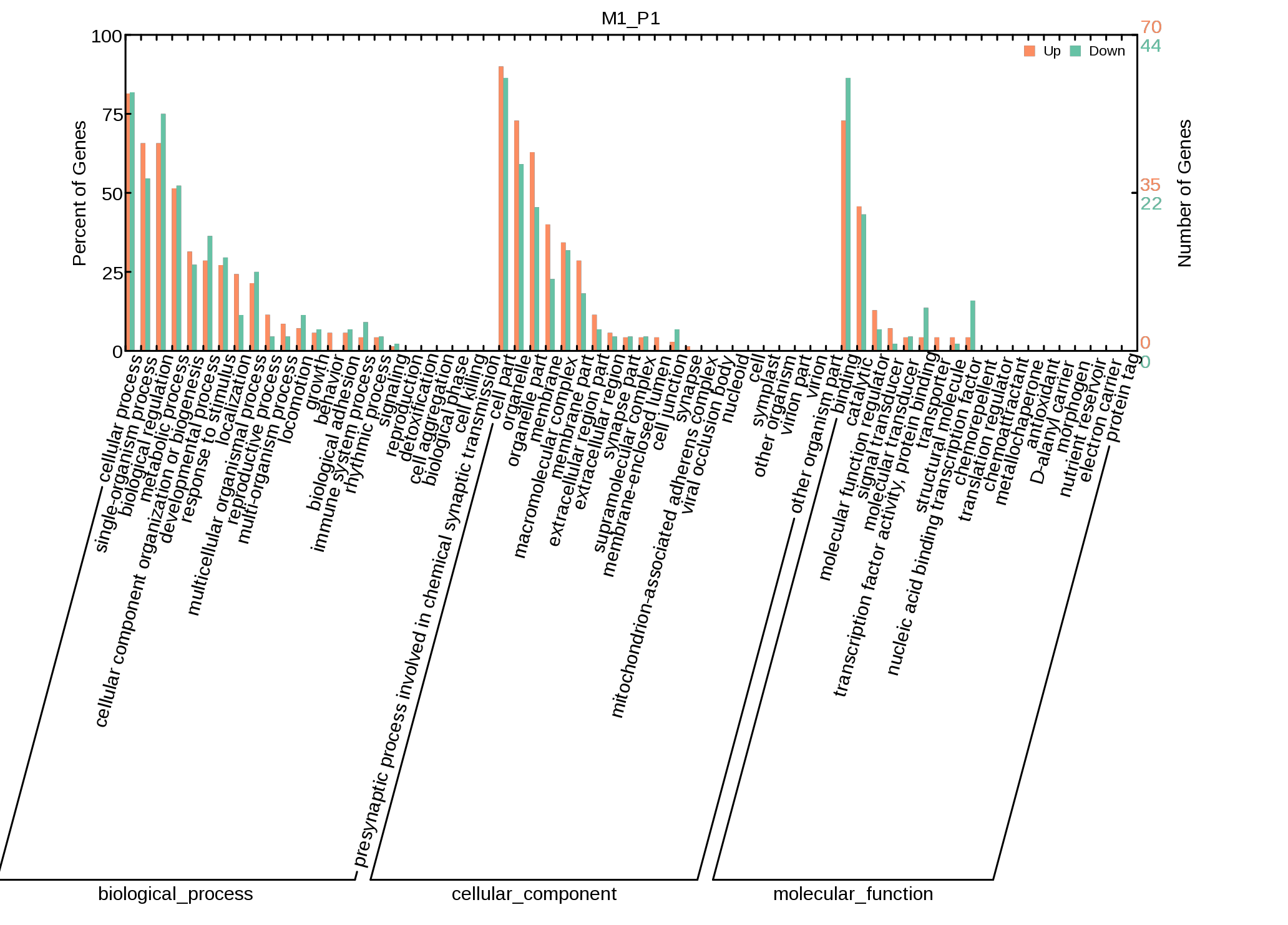

Supplement: Supplementary Figure 1 — Volcano map of different groups of differential RNAs. (A–C) Expression profiles of mRNAs and LncRNAs (M1 vs. P1, M2 vs. P2, M3 vs. P3). (D–F) Expression profiles of circRNAs (M1 vs. P1, M2 vs. P2, M3 vs. P3). Green points represent down-regulated RNAs; red points represent up-regulated RNAs; gray points represent not significantly expressed RNAs in the volcano plots. (G–I) Expression profiles of miRNAs (M1 vs. P1, M2 vs. P2, M3 vs. P3). Blue points represent down-regulated RNAs; yellow points represent up-regulated RNAs; gray points represent not significantly expressed RNAs in the volcano plots. X-axis: Fold change log2 ratio of RNAs. Y-axis: false discovery rate values (-log10 transformed). [file Data_Sheet_1.ZIP › Supplementary figures and tables/FigureS3C.tif]

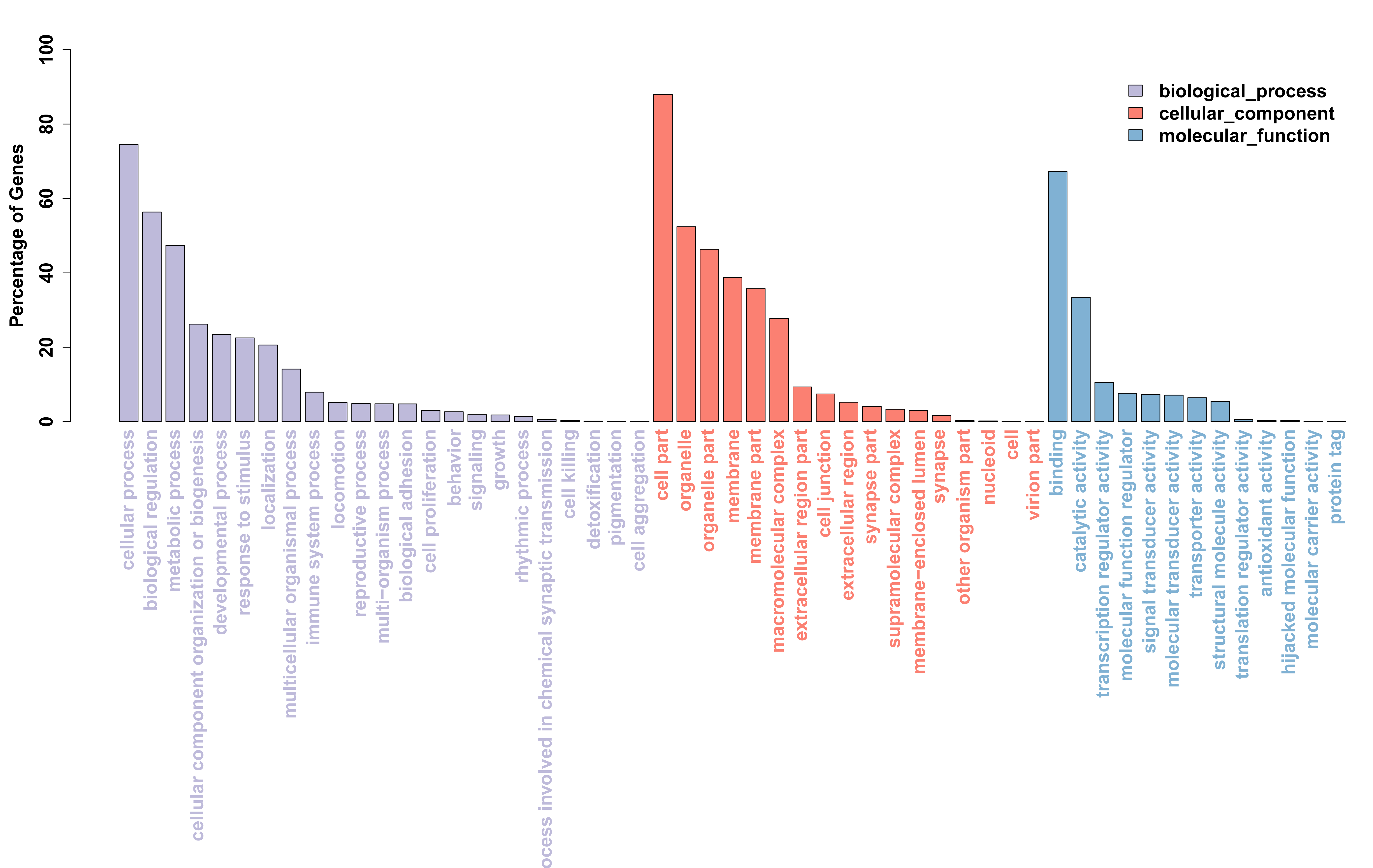

Supplement: Supplementary Figure 1 — Volcano map of different groups of differential RNAs. (A–C) Expression profiles of mRNAs and LncRNAs (M1 vs. P1, M2 vs. P2, M3 vs. P3). (D–F) Expression profiles of circRNAs (M1 vs. P1, M2 vs. P2, M3 vs. P3). Green points represent down-regulated RNAs; red points represent up-regulated RNAs; gray points represent not significantly expressed RNAs in the volcano plots. (G–I) Expression profiles of miRNAs (M1 vs. P1, M2 vs. P2, M3 vs. P3). Blue points represent down-regulated RNAs; yellow points represent up-regulated RNAs; gray points represent not significantly expressed RNAs in the volcano plots. X-axis: Fold change log2 ratio of RNAs. Y-axis: false discovery rate values (-log10 transformed). [file Data_Sheet_1.ZIP › Supplementary figures and tables/FigureS3D.tif]

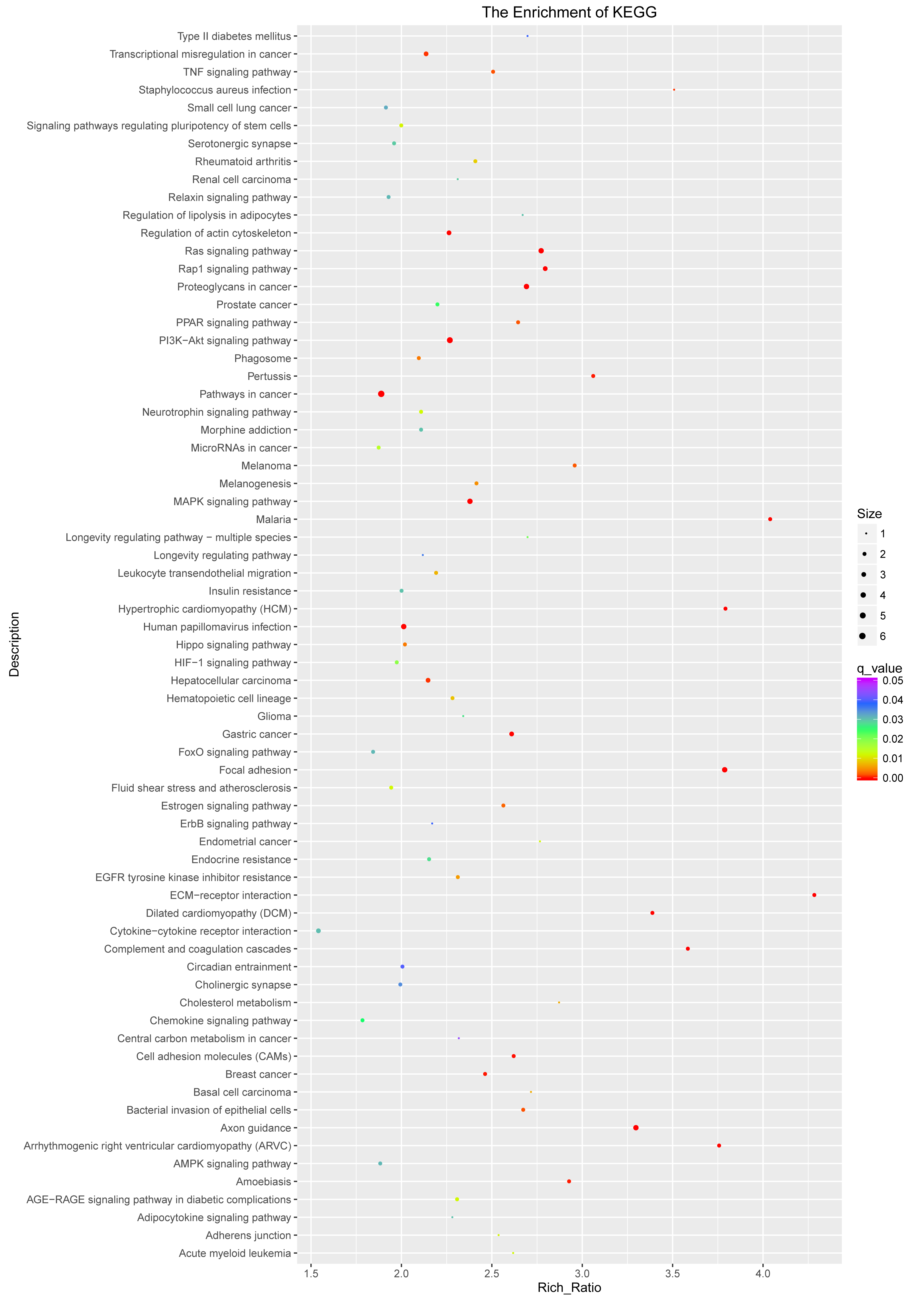

Supplement: Supplementary Figure 1 — Volcano map of different groups of differential RNAs. (A–C) Expression profiles of mRNAs and LncRNAs (M1 vs. P1, M2 vs. P2, M3 vs. P3). (D–F) Expression profiles of circRNAs (M1 vs. P1, M2 vs. P2, M3 vs. P3). Green points represent down-regulated RNAs; red points represent up-regulated RNAs; gray points represent not significantly expressed RNAs in the volcano plots. (G–I) Expression profiles of miRNAs (M1 vs. P1, M2 vs. P2, M3 vs. P3). Blue points represent down-regulated RNAs; yellow points represent up-regulated RNAs; gray points represent not significantly expressed RNAs in the volcano plots. X-axis: Fold change log2 ratio of RNAs. Y-axis: false discovery rate values (-log10 transformed). [file Data_Sheet_1.ZIP › Supplementary figures and tables/FigureS4A.tif]

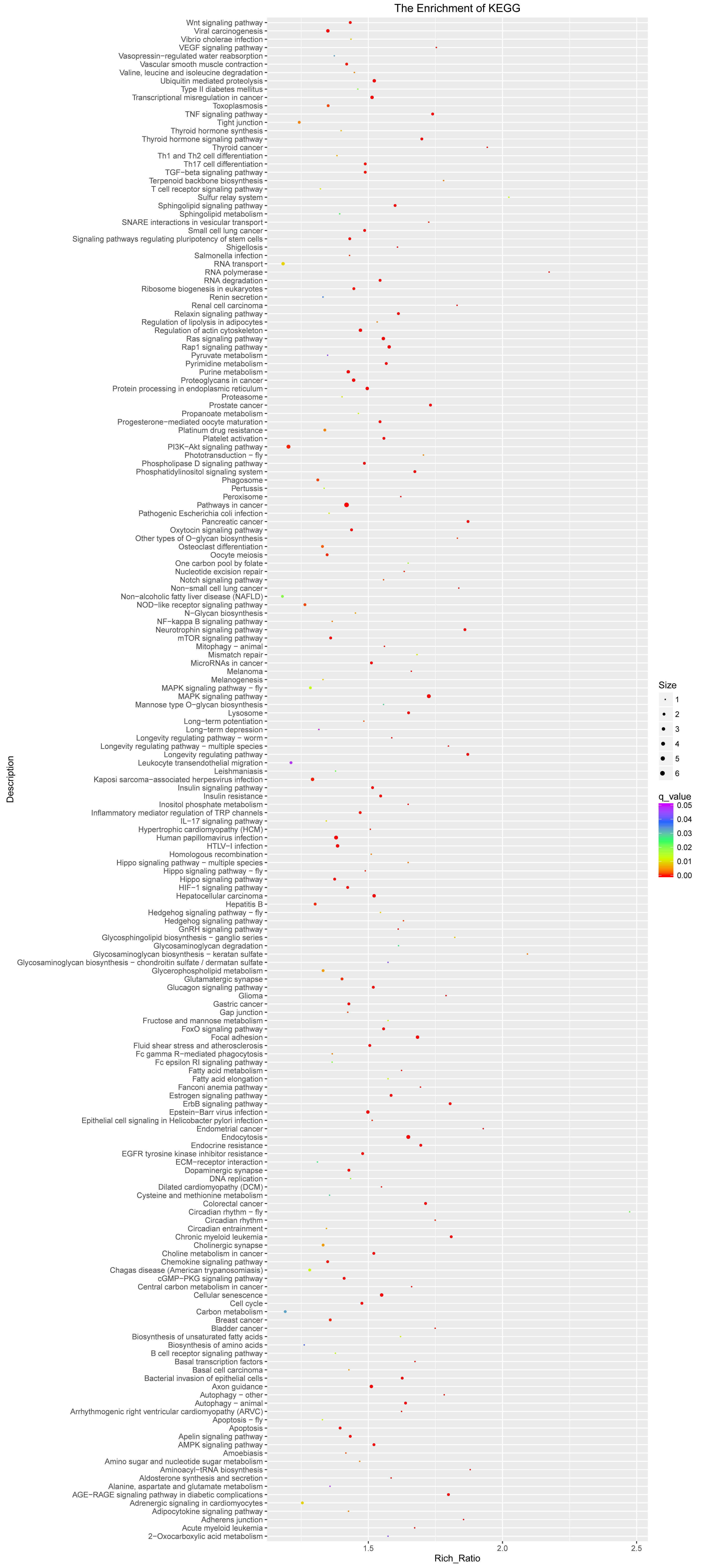

Supplement: Supplementary Figure 1 — Volcano map of different groups of differential RNAs. (A–C) Expression profiles of mRNAs and LncRNAs (M1 vs. P1, M2 vs. P2, M3 vs. P3). (D–F) Expression profiles of circRNAs (M1 vs. P1, M2 vs. P2, M3 vs. P3). Green points represent down-regulated RNAs; red points represent up-regulated RNAs; gray points represent not significantly expressed RNAs in the volcano plots. (G–I) Expression profiles of miRNAs (M1 vs. P1, M2 vs. P2, M3 vs. P3). Blue points represent down-regulated RNAs; yellow points represent up-regulated RNAs; gray points represent not significantly expressed RNAs in the volcano plots. X-axis: Fold change log2 ratio of RNAs. Y-axis: false discovery rate values (-log10 transformed). [file Data_Sheet_1.ZIP › Supplementary figures and tables/FigureS4B.tif]

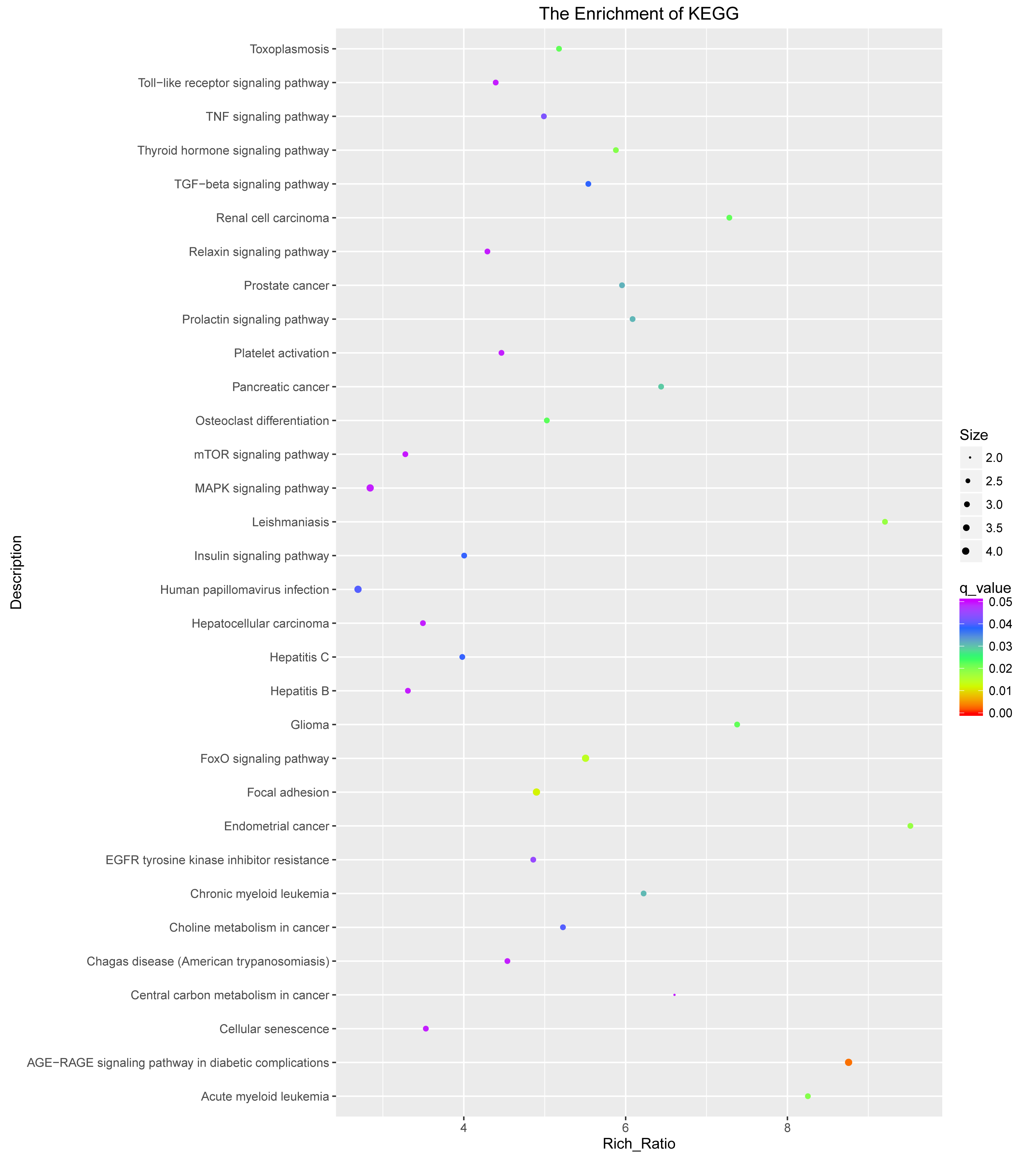

Supplement: Supplementary Figure 1 — Volcano map of different groups of differential RNAs. (A–C) Expression profiles of mRNAs and LncRNAs (M1 vs. P1, M2 vs. P2, M3 vs. P3). (D–F) Expression profiles of circRNAs (M1 vs. P1, M2 vs. P2, M3 vs. P3). Green points represent down-regulated RNAs; red points represent up-regulated RNAs; gray points represent not significantly expressed RNAs in the volcano plots. (G–I) Expression profiles of miRNAs (M1 vs. P1, M2 vs. P2, M3 vs. P3). Blue points represent down-regulated RNAs; yellow points represent up-regulated RNAs; gray points represent not significantly expressed RNAs in the volcano plots. X-axis: Fold change log2 ratio of RNAs. Y-axis: false discovery rate values (-log10 transformed). [file Data_Sheet_1.ZIP › Supplementary figures and tables/FigureS4C.tif]

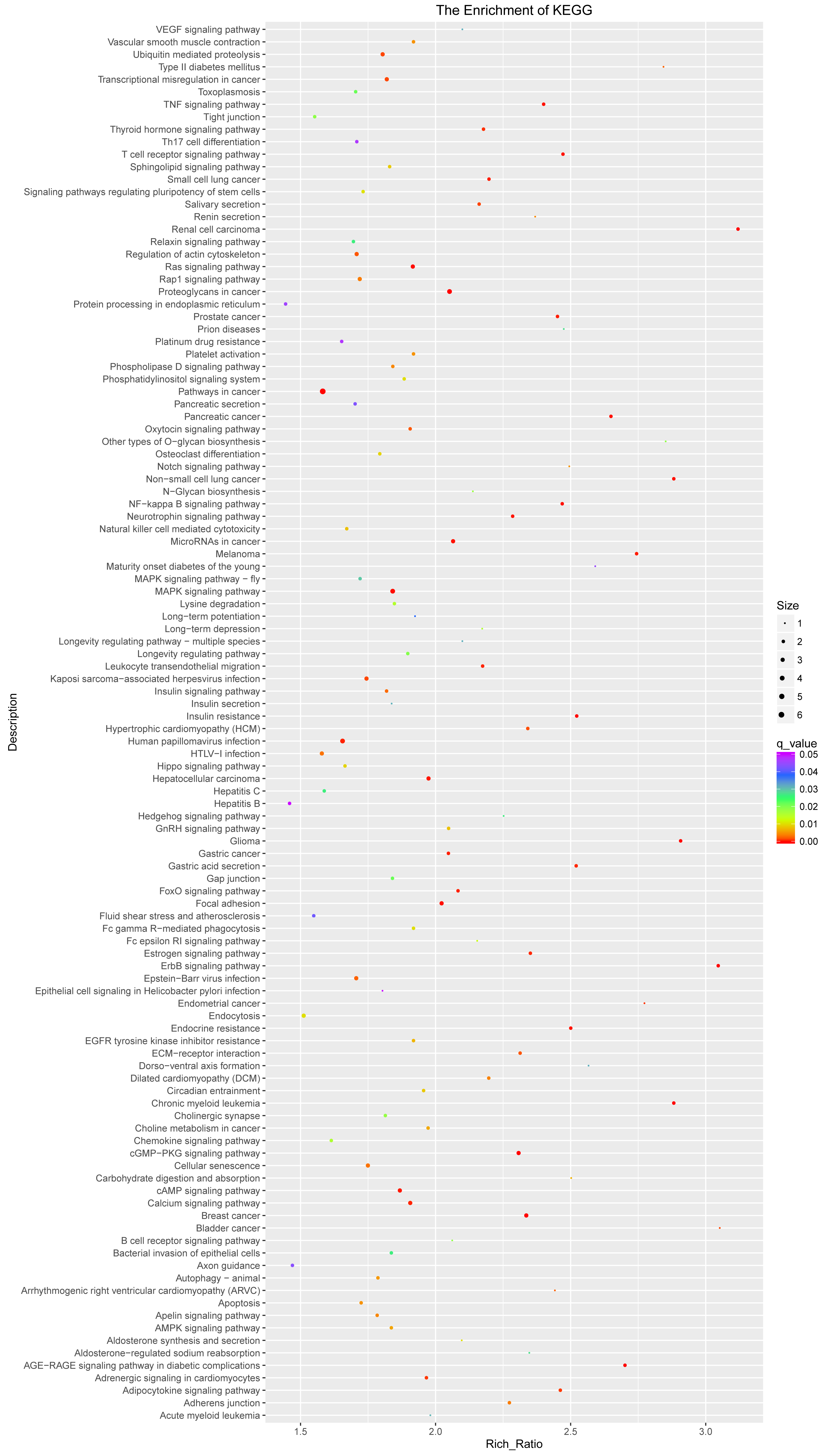

Supplement: Supplementary Figure 1 — Volcano map of different groups of differential RNAs. (A–C) Expression profiles of mRNAs and LncRNAs (M1 vs. P1, M2 vs. P2, M3 vs. P3). (D–F) Expression profiles of circRNAs (M1 vs. P1, M2 vs. P2, M3 vs. P3). Green points represent down-regulated RNAs; red points represent up-regulated RNAs; gray points represent not significantly expressed RNAs in the volcano plots. (G–I) Expression profiles of miRNAs (M1 vs. P1, M2 vs. P2, M3 vs. P3). Blue points represent down-regulated RNAs; yellow points represent up-regulated RNAs; gray points represent not significantly expressed RNAs in the volcano plots. X-axis: Fold change log2 ratio of RNAs. Y-axis: false discovery rate values (-log10 transformed). [file Data_Sheet_1.ZIP › Supplementary figures and tables/FigureS4D.tif]
